# COVID-19 generates hyaluronan fragments that directly induce endothelial barrier dysfunction

**DOI:** 10.1172/jci.insight.147472

**Published:** 2021-09-08

**Authors:** Kimberly A. Queisser, Rebecca A. Mellema, Elizabeth A. Middleton, Irina Portier, Bhanu Kanth Manne, Frederik Denorme, Ellen J. Beswick, Matthew T. Rondina, Robert A. Campbell, Aaron C. Petrey

**Affiliations:** 1 University of Utah Molecular Medicine Program, Salt Lake City, Utah, USA.; 2 Department of Pathology and; 3 Division of General Internal Medicine, Department of Internal Medicine, University of Utah School of Medicine, Salt Lake City, Utah, USA.; 4 Division of Gastroenterology, Department of Internal Medicine, University of Utah, Salt Lake City, Utah, USA.; 5 Geriatric Research, Education, and Clinical Center and; 6 Department of Internal Medicine, George E. Wahlen Salt Lake City Veterans Affairs Medical Center, Salt Lake City, Utah, USA.

**Keywords:** COVID-19, Vascular Biology, Extracellular matrix, Glycobiology

## Abstract

Vascular injury has emerged as a complication contributing to morbidity in coronavirus disease 2019 (COVID-19). The glycosaminoglycan hyaluronan (HA) is a major component of the glycocalyx, a protective layer of glycoconjugates that lines the vascular lumen and regulates key endothelial cell functions. During critical illness, as in the case of sepsis, enzymes degrade the glycocalyx, releasing fragments with pathologic activities into circulation and thereby exacerbating disease. Here, we analyzed levels of circulating glycosaminoglycans in 46 patients with COVID-19 ranging from moderate to severe clinical severity and measured activities of corresponding degradative enzymes. This report provides evidence that the glycocalyx becomes significantly damaged in patients with COVID-19 and corresponds with severity of disease. Circulating HA fragments and hyaluronidase, 2 signatures of glycocalyx injury, strongly associate with sequential organ failure assessment scores and with increased inflammatory cytokine levels in patients with COVID-19. Pulmonary microvascular endothelial cells exposed to COVID-19 milieu show dysregulated HA biosynthesis and degradation, leading to production of pathological HA fragments that are released into circulation. Finally, we show that HA fragments present at high levels in COVID-19 patient plasma can directly induce endothelial barrier dysfunction in a ROCK- and CD44-dependent manner, indicating a role for HA in the vascular pathology of COVID-19.

## Graphical abstract

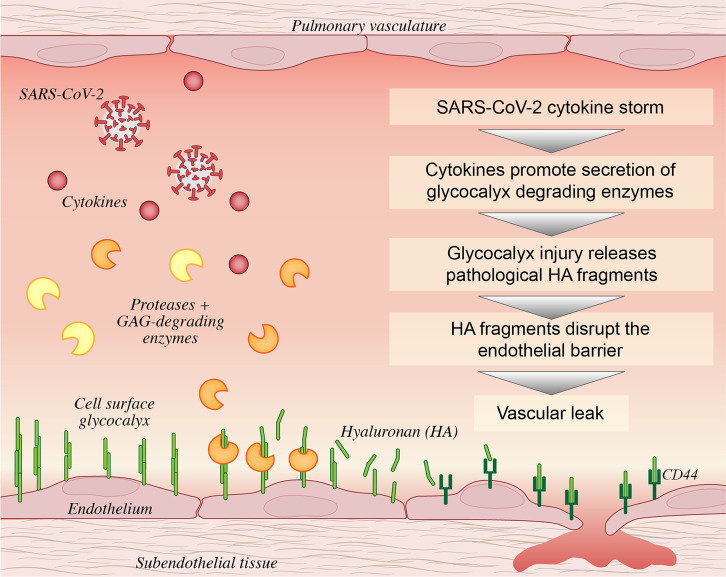

## Introduction

The severe acute respiratory syndrome (SARS) coronavirus disease 2019 (COVID-19) is a crippling public health crisis caused by SARS coronavirus 2 (SARS-CoV-2). COVID-19 is responsible for > 49.7 million cases and 1.2 million deaths globally (1). An exacerbated immune response (2, 3) is strongly implicated as driving damage to the airspaces and extrapulmonary symptoms, including acute kidney injury (4, 5), acute cardiac injury (6), coagulopathy (7), thrombosis (8), platelet hyperreactivity (9, 10), and circulatory shock (11). Patients with cardiovascular risk factors such as obesity, diabetes, and hypertension are at increased risk of severe complications (12–14), including acute respiratory distress syndrome (ARDS), and chronic vascular endothelial injury is often present in patients with these comorbidities. Findings of endotheliopathy (15), evidence of SARS-CoV-2 infection of pulmonary (16–18) and extrapulmonary (19) endothelial cells (ECs), reports of viremia (20, 21), and multiorgan injury lead to the hypothesis that COVID-19 is, in part, a vascular illness.

Under healthy conditions, the endothelium possesses endogenous antiinflammatory, anticoagulant, and vasodilatory mechanisms that balance factors that promote inflammation, coagulation, and vasoconstriction. However, in aged individuals and those with cardiovascular disease such as diabetes and hypertension, the 2 most prevalent comorbidities in COVID-19, homeostatic endothelial functions become impaired (22). Clinical studies provide evidence that endothelial dysfunction is a major determinant of severe COVID-19, and patients have markers of endothelial injury, including increased levels of fibrinogen, fibrin degradation products, D-dimer, von Willebrand factor (23, 24), and soluble thrombomodulin (15), as well as signatures of impaired endothelial function such as apoptotic ECs (17), decreased nitric oxide availability (25), and vascular leakage (26).

At the vascular surface, the glycocalyx plays an essential role in the regulation of barrier integrity, nitric oxide production and vasorelaxation, resistance to oxidative stress, coagulation, and inflammation (27, 28). The glycocalyx is a complex network composed of the glycosaminoglycans (GAGs) hyaluronan (HA), heparan sulfate (HS), chondroitin sulfate (CS) and other glycoconjugates at the interface between the cell surface and the extracellular environment. The glycocalyx undergoes constitutive remodeling, and homeostasis is maintained by a balance of synthesis and degradation. However, infection, inflammation, ischemia/reperfusion, and hyperglycemia lead to destruction of the glycocalyx and release of bioactive fragments, which exacerbate disease (29, 30). In bacterial sepsis, glycocalyx degradation associates with the presence and severity of disease and is a causative element in recruitment of immune cells, barrier disruption, and development of ARDS (31–34). The glycocalyx is degraded at the cell surface by the activity of endo-β-glucuronidases, which regulate the availability of HA or HS chains for receptor binding by cleaving these polymers and thereby controlling several important ligand-binding interactions with known roles in leukocyte recruitment, neutrophil function, endothelial behavior, and cytokine and growth factor release (33, 35–39). Hyaluronidases predominantly degrade HA, while heparanase predominantly degrades HS; both exhibit limited activity toward CS, which has no known specific degradation enzyme in mammals (40, 41). These enzymes are primarily regulated at the transcript level and expressed in many cell types, including ECs, leukocytes, mast cells, and platelets. Degradation products of HA and HS act as endogenous danger signals capable of amplifying proinflammatory responses both in vitro and in murine models of disease (42–45). Recent reports suggest that endothelial damage and glycocalyx injury are present in COVID-19 (46–48). However, whether direct viral infection or cytokine release are the underlying mechanisms leading to glycocalyx injury, and how glycocalyx fragments contribute to the pathology of COVID-19, are not known.

In this prospective study, we examined the concentrations of circulating GAGs and the activities of GAG-degrading enzymes in acutely ill patients with COVID-19 compared with patients with sepsis and matched healthy donors. Analysis of plasma isolated from SARS-CoV-2–infected patients revealed significantly increased concentrations of circulating GAGs, and this was accompanied with elevated hyaluronidase and heparanase activities. Circulating HA and HS fragments associate with severity of disease, and HA and hyaluronidase were also associated with clinical parameters and concentrations of plasma cytokines. Stimulation of human pulmonary microvascular ECs with plasma from patients with COVID-19 promotes release of HA into culture media, increased hyaluronidase activity, and transcriptional activation of HA biosynthesis and degradation when compared with plasma from healthy donors. Analysis of circulating HA isolated from patients, and HA from cells exposed to COVID-19 patient plasma, demonstrates that pathological HA degradation products are significantly increased during disease. Finally, treatment of ECs with HA fragments purified from the plasma of infected patients, but not HA from healthy donors, promotes disruption of endothelial barrier integrity in a Rho-associated protein kinase (ROCK) and CD44-dependent manner. Our data provide evidence that SARS-CoV-2 infection is characterized by degradation of the endothelial glycocalyx, that COVID-19 cytokine milieu stimulates aberrant synthesis and degradation of HA in pulmonary ECs, and that HA fragments present at high concentrations in COVID-19 patient plasma are capable of directly mediating endothelial dysfunction.

## Results

### COVID-19 patient cohort.

Patients with COVID-19 were matched by age, sex, and race to critically ill patients with sepsis and to healthy donors (Table 1). SARS-CoV-2 virus was uniformly detected in patients diagnosed with SARS-CoV-2 by PCR. Approximately 36% of patients with COVID-19 were in the ICU and 35% required mechanical ventilation due to respiratory failure. Hospitalized non-ICU and ICU patients with COVID-19 had comorbidities of hypertension, diabetes, and obesity, consistent with previous observations (13). Mortality rate was ~17% among all patients with COVID-19, consistent with hospitalized mortality rates (13.4%) in patients with COVID-19 in Utah per the Utah Department of Health as of November 8, 2020.

### Circulating GAGs are increased in patients with COVID-19 and associate with disease severity.

**Figure 1:**
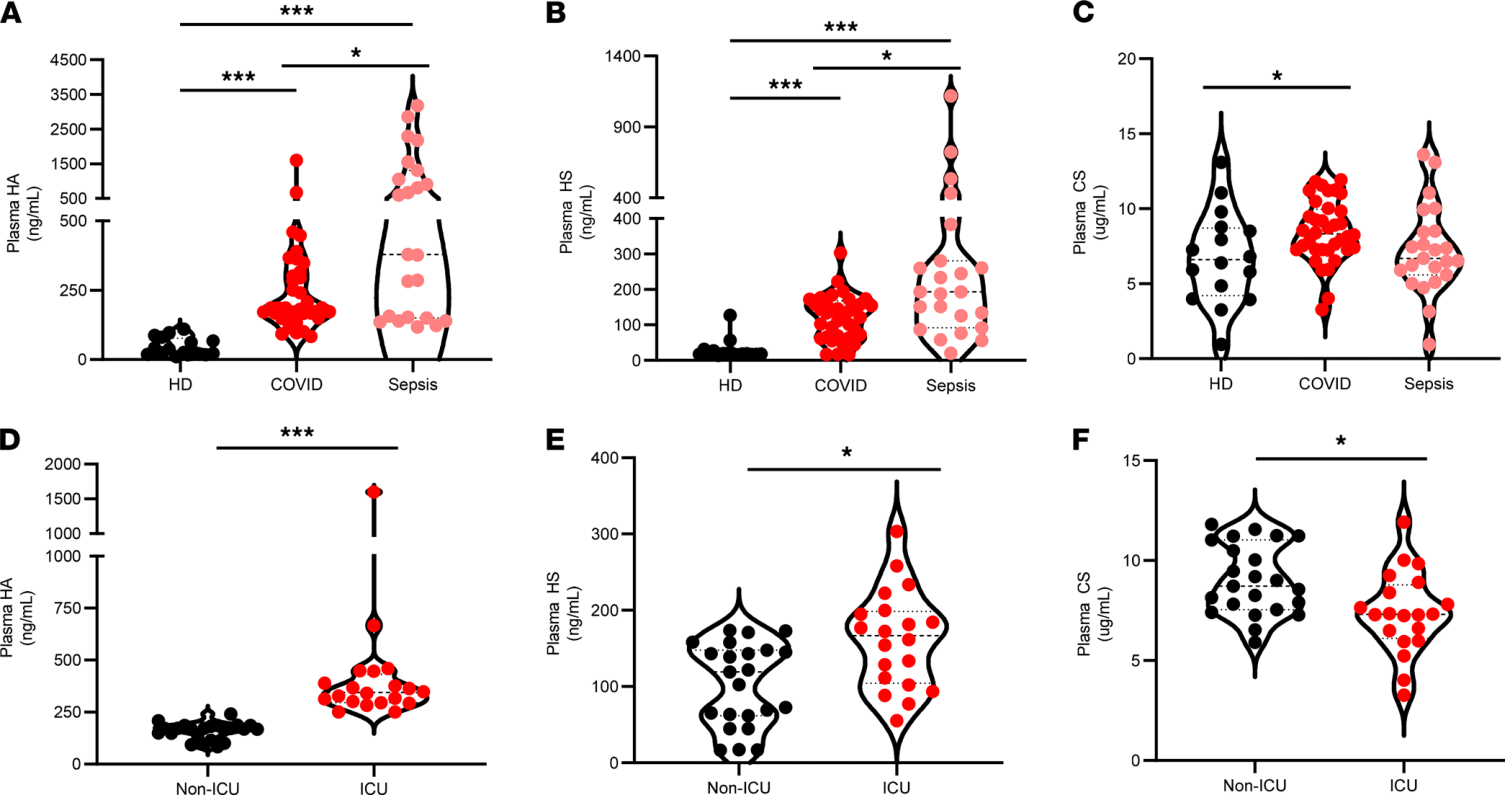
Circulating glycosaminoglycans are increased in patients with COVID-19 and associate with disease severity. Circulating glycosaminoglycans were measured in plasma collected from patients with COVID-19 (*n* = 46) and patients with sepsis (*n* = 23) within 72 hours of ICU admission or age-, race-, and sex-matched healthy donors (*n* = 18) as described in Methods. (**A**–**C**) Levels of HA (**A**), HS (**B**), and CS (**C**) in COVID-19 compared with healthy patients and patients with sepsis. (**D**–**F**) Comparison of HA (**D**), HS (**E**), and CS (**F**) levels by disease status in ICU- (*n* = 20) and non-ICU–admitted (*n* = 23) patients with COVID-19. A thick dashed line indicates the median, and thin dashed lines indicate either quartile. Data are reported as mean ± SEM; *P < 0.05 and ***P < 0.001. Differences between multiple groups were calculated using 1-way ANOVA (Kruskal-Wallis test) with Dunn’s correction for multiple comparisons.

Endothelial glycocalyx degradation has been increasingly implicated in the pathogenesis of critical illnesses, including as sepsis and influenza (49). To determine the extent of injury to the endothelial glycocalyx in COVID-19, we quantified circulating HA, HS, and CS in plasma from 36 patients with COVID-19 (23 non-ICU, 13 ICU) and 18 matched healthy donors recruited from the greater Salt Lake City area (Utah, USA). As shown in Figure 1, A–C, this analysis revealed significant increases in mean plasma concentrations of HA (5.8-fold), HS (4.3-fold), and CS (1.3-fold) in patients with COVID-19 in comparison with healthy donors. To evaluate whether the levels of circulating GAGs in COVID-19 were comparable with other critical illnesses, we compared our COVID-19 patient cohort with patients with clinical sepsis (*n* = 23; Figure 1, A–C). We observed that plasma concentrations of HA and HS are significantly increased in plasma from patients with sepsis (9.3-fold and 9.2-fold, respectively) when compared with healthy donors and are also increased in patients with sepsis as compared with measurements in patients with COVID-19, but CS concentrations were not significantly different. Because patients with COVID-19 — as well as patients with sepsis — in our cohort were treated with derivatives of heparin, a highly sulfated form of HS, we evaluated whether the treatment might confound accurate detection of HS. We observed that the addition of pharmacologic concentrations of enoxaparin to patient plasma had no significant influence on detection of HS levels in patients (Supplemental Figure 1; supplemental material available online with this article; https://doi.org/10.1172/jci.insight.147472DS1). We next evaluated whether circulating GAG levels associate with COVID-19 disease severity and found that circulating levels of HA and HS were significantly increased in ICU-admitted patients, while CS levels were decreased compared with non-ICU admitted patients (Figure 1, D–F). Taken together, these data suggest that wide-spread endothelial glycocalyx injury is a characteristic of COVID-19 and that mean concentrations of HA and HS in ICU-admitted patients are comparable with circulating concentrations detected in patients with sepsis.

### Glycocalyx-degrading enzyme activities are elevated in patients with COVID-19.

**Figure 2:**
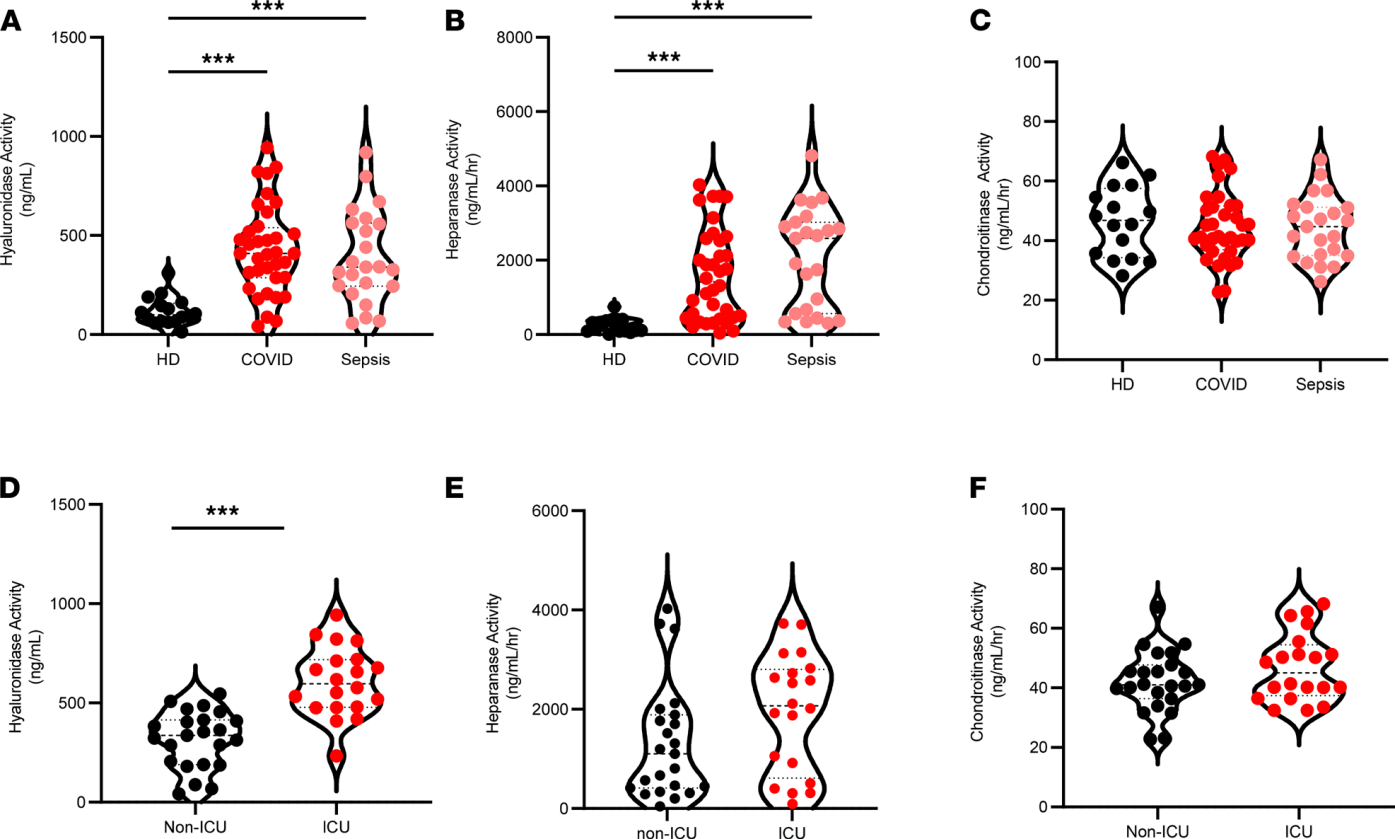
Glycocalyx-degrading enzyme activities are elevated in patients with COVID-19. Activity of glycosaminoglycan-degrading enzymes were measured in plasma collected from patients with COVID-19 (*n* = 46) and patients with sepsis (*n* = 23) within 72 hours of ICU admission or age-, race-, and sex-matched healthy donors (*n* = 18). (**A**–**C**) Level of enzymatic activity degrading HA (**A**), HS (**B**), and CS (**C**) in COVID-19 compared with normal and septic patients. (**D**–**F**) Comparison of enzymatic activity degrading HA (**D**), HS (**E**), and CS (**F**) levels by disease status in ICU- (n = 20) and non–ICU-admitted (n = 23) patients with COVID-19. A thick dashed line indicates the median, and thin dashed lines indicate either quartile. Data are reported as mean ± SEM; **P < 0.01 and ***P < 0.001. Differences between multiple groups were calculated using 1-way ANOVA (Kruskal-Wallis test) with Dunn’s correction for multiple comparisons.

The increased concentrations of circulating GAGs observed in COVID-19 are suggestive of increased activity of enzymes known to release GAG fragments into circulation. We therefore measured hyaluronidase, heparanase, and chondroitinase activities in plasma of patients with COVID-19 compared with either healthy controls or patients with sepsis. Plasma activities of hyaluronidase and heparanase were significantly elevated in both COVID-19 and patients with sepsis compared with healthy controls (Figure 2, A and B), and no difference in chondroitinase activity was observed between patient groups (Figure 2C). We next compared activity levels of GAG-degrading enzymes in non-ICU– and ICU-admitted patients with COVID-19 and observed that ICU-admitted patients show elevated circulating hyaluronidase (Figure 2D) and heparanase (Figure 2E), as well as a trend toward increase in chondroitinase activity (Figure 2F). Only hyaluronidase levels were found to be significantly different between ICU- and non-ICU patients (Figure 2D). In addition to direct degradation of GAGs, the activated endothelium may also release proteases contained within Weibel-Palade bodies and secretory lysosomes that promote proteolytic shedding of proteoglycan core proteins. We therefore measured the activities of matrix metalloproteinases (MMP2/9) and cathepsin D — proteases with known roles in endothelial injury (50) — and found that MMP (Supplemental Figure 2A) and cathepsin D (Supplemental Figure 2B) activities are significantly increased in patients with COVID-19 compared with healthy donors.

### Circulating HA and hyaluronidase activity correlates with clinical and inflammatory signatures of COVID-19.

**Figure 3:**
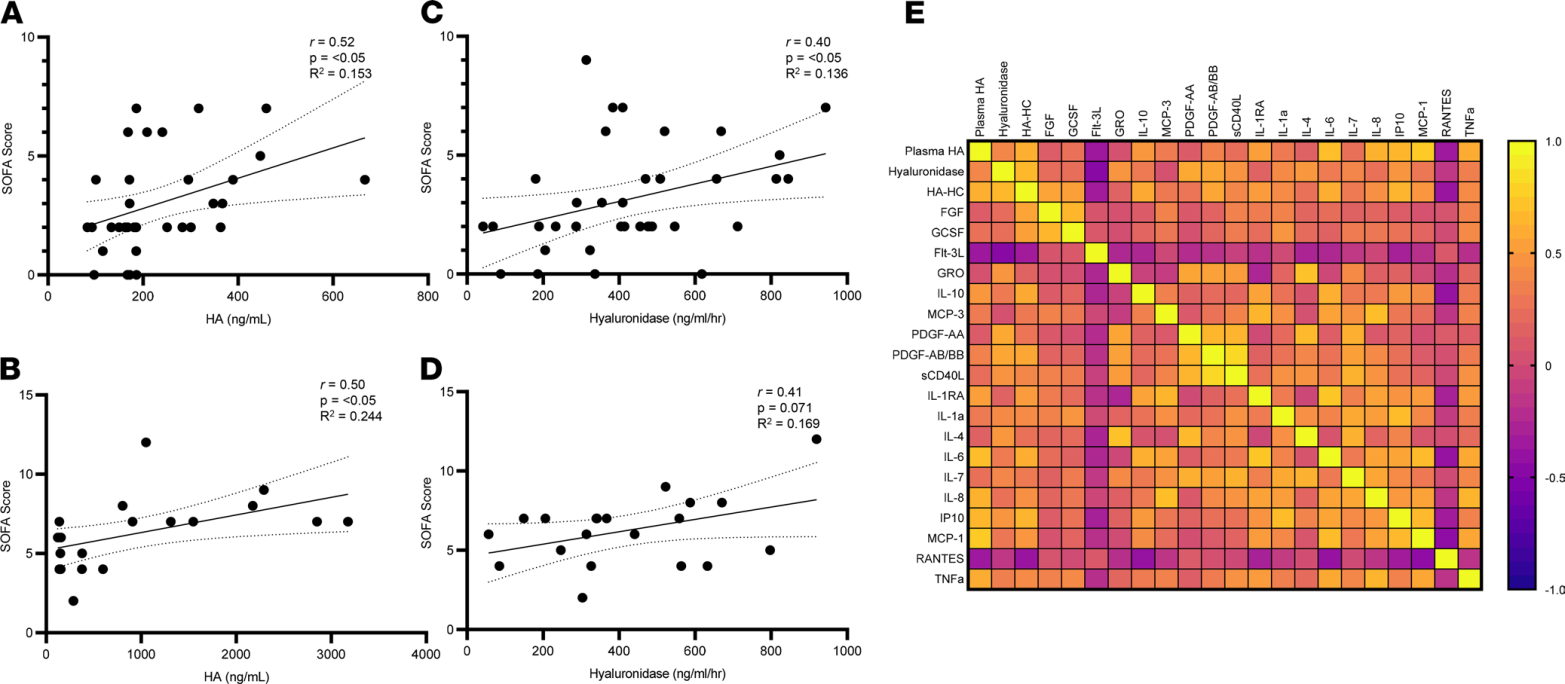
Circulating levels of HA and hyaluronidase activity correlate with clinical and inflammatory signatures of COVID-19. (**A**–**D**) Correlation analysis severity of sequential organ failure assessment (SOFA) scores with plasma HA concentration and hyaluronidase activity levels in COVID-19 (*n* = 46) (**A** and **C**) and sepsis (*n* = 23) (**B** and **D**) patients. (**E**) Spearman correlation matrix of plasma HA, hyaluronidase (HA’ase) activity, and cytokines in patients with COVID-19 (*n* = 46) demonstrated to be statistically significant (*P < 0.05) between healthy controls and patients with COVID-19. Yellow indicates a positive correlation, and purple indicates a negative correlation. Pearson correlation coefficient was used to determine the *r* value of the correlation between the 2 groups.

Circulating GAGs have been shown to associate with lung injury (32), clinical parameters in ARDS (51, 52), and other forms of organ injury in sepsis (53, 54). We therefore evaluated whether circulating levels of GAGs or degrading enzyme activities associate with clinical assessments in patients with COVID-19. We found that plasma HA levels show a significant positive correlation with sequential organ failure assessment (SOFA) score, an index of illness severity in patients with COVID-19 (Figure 3A) and in patients with sepsis (Figure 3B), similar to previous reports (55). Similarly, hyaluronidase activity also was found to have a significant positive correlation with SOFA score in patients with COVID-19 (Figure 3C) and trended toward significance in patients with sepsis (Figure 3D). We found no correlation with severity of illness for HS, CS, heparanase, or chondroitinase activity in patients with COVID-19 (Supplemental Figure 3, A–C), though HS shows a strong positive association in our septic patient population (Supplemental Figure 3C) similar to previous reports (32, 33). We next performed intercorrelation analysis between plasma HA levels and hyaluronidase activity with previously published plasma cytokine levels (56) that we determined to be significantly increased in our patients with COVID-19 compared with healthy controls (Figure 3E and Supplemental Table 1). This analysis shows that plasma levels of HA and hyaluronidase in patients with COVID-19 each are correlated with inflammatory cytokines known to have roles in either HA synthesis or degradation such as IL-6, IL-8, MCP-1, TNF-α, and IP10. Regulation of HA synthesis and degradation are typically inversely associated. Unexpectedly, of all 19 cytokines compared, only IP10, a biomarker and mediator of Kawasaki disease pathology (57), shows a significant positive correlation with both HA and hyaluronidase in patients with COVID-19 but not in healthy donors (Supplemental Figure 3D and Supplemental Table 2). Interestingly, some children infected with SARS-CoV-2 develop a severe inflammatory disease with characteristics of Kawasaki disease (58).

### Treatment with COVID-19 plasma induces HA synthesis and degradation in lung microvascular ECs.

**Figure 4:**
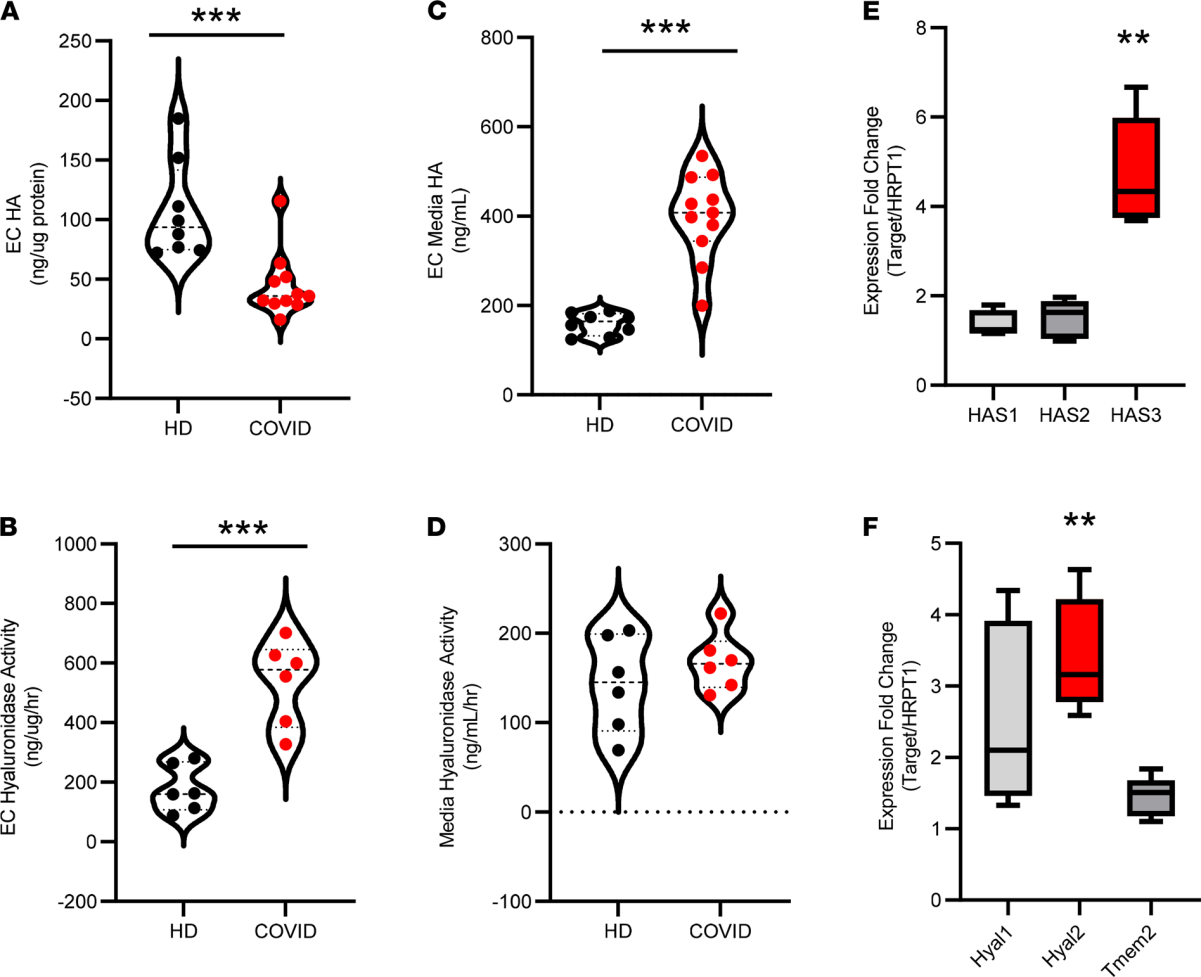
COVID-19 plasma induces HA synthesis and degradation in lung microvascular ECs. Primary human pulmonary microvascular endothelial cells were exposed to 1.5% plasma from patients with COVID-19 or healthy donors and measured for changes in HA biosynthesis and degradation. (**A**–**D**) After 16 hours, levels of HA and hyaluronidase activity associated with the cell layer (**A** and **B**) or in conditioned media (**C** and **D**) were measured, and levels present in 1.5% plasma alone was subtracted as background. (**E** and **F**) qPCR of HA biosynthetic (**E**) and HA degrading (**F**) enzyme transcript levels in cells treated with either COVID-19 or control plasma for 2 hours. In all figures, a thick dashed line indicates the median and additional thin dashed lines indicate either quartile. Data are reported as mean ± SEM; **P < 0.01 and ***P < 0.001, using unpaired Student’s *t* test, 2 tailed, with at least 5 independent experiments of at least 5 patients each. Circles represent individual patient plasma samples.

Based on our observation that cytokines present in patients with COVID-19 associate with circulating HA and hyaluronidase levels, we next explored whether plasma from infected patients might promote dysregulated synthesis and degradation of HA in human lung microvascular ECs (LMVECs). We found that treatment of cultured ECs with plasma from patients with COVID-19 led to decreased cellular HA (Figure 4A) and increased cell-associated hyaluronidase activity (Figure 4B) when compared with cells treated with healthy donor plasma and normalized against plasma alone. Analysis of media from cells exposed to COVID-19 plasma revealed a corresponding increase in HA (Figure 4C) but not hyaluronidase activity (Figure 4D), as compared with cells treated with healthy plasma. We examined the media of treated LMVECs for sheddase activity that might be induced by plasma cytokines and observed increased MMP2/9 and cathepsin D activities in the media of cells exposed to COVID-19 plasma compared with healthy controls when normalized against plasma alone, suggesting release by ECs themselves (Supplemental Figure 2, C and D). We next asked whether plasma from patients with COVID-19 could alter transcription of genes responsible for HA synthesis or degradation and found that treatment with COVID-19 patient plasma significantly increases mRNA expression of HA synthase-3 (HAS3), hyaluronidase-1 (HYAL-1), and HYAL-2 compared with treatment with healthy donor plasma (Figure 4, E and F). Given the reports of SARS-CoV-2 infection of ECs, we next sought to determine whether the observed activation of ECs by COVID-19 plasma was due to activation by humoral factors or by infection with SARS-CoV-2 that might be present in COVID-19 plasma samples. Examination of ECs cultured in the presence of COVID-19 plasma indicates that transcripts corresponding to the SARS-CoV-2 N1 gene are undetectable as compared with tracheal aspirates from patients with COVID-19 (Supplemental Figure 4). These findings suggest that cytokines associated with COVID-19, rather than only direct viral infection, promote dysregulated biosynthesis and degradation of endothelial HA by the pulmonary microvasculature.

### Circulating HA is present as pathological low–molecular weight fragments in COVID-19 patient plasma.

**Figure 5:**
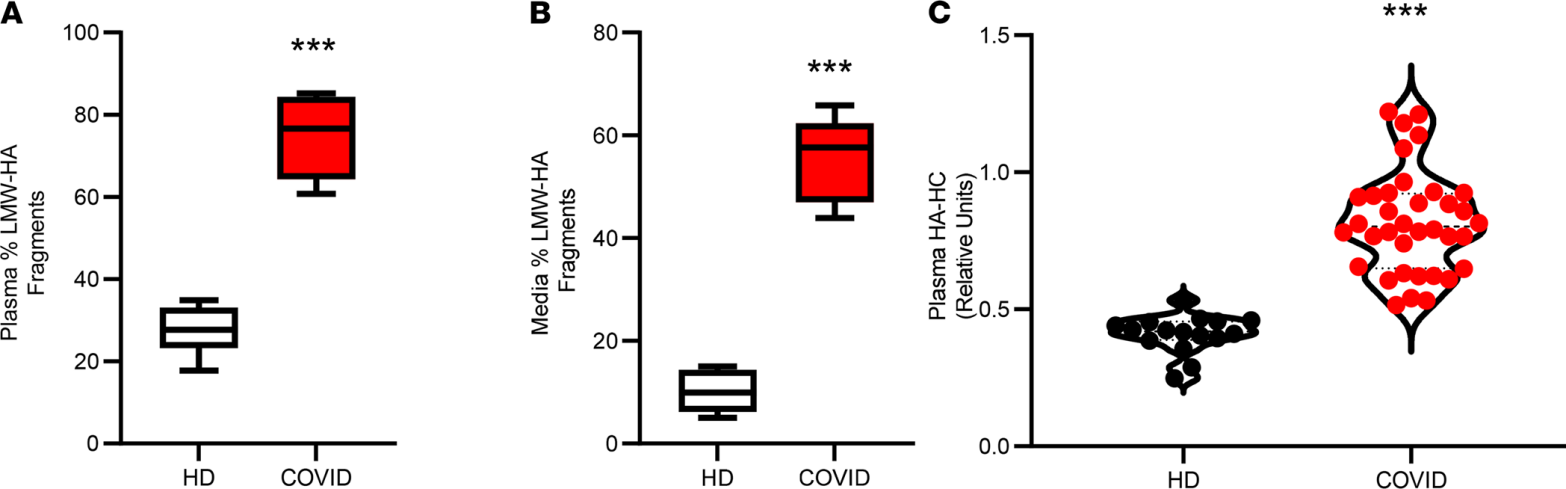
Circulating HA is present as pathological low–molecular weight fragments. The molecular weight profile of HA was fractionated stepwise and measured by competitive ELISA assay. (**A** and **B**) HA from patient plasma (**A**) or pulmonary microvascular endothelial cells stimulated with either healthy donor or COVID-19 patient plasmas (**B**) is reported as the relative abundance of LMW-HA fragments (<50 kDa, *n* = 5 patients each). (**C**) ELISA measurement of HA-HC in plasma from patients with COVID-19 (*n* = 46) or healthy donors (*n* = 18). A thick dashed line indicates the median, and thin dashed lines indicate either quartile. Data are reported as mean ± SEM; ***P < 0.001, using unpaired Student’s *t* test, 2 tailed.

Many of the biological responses governed by HA are dependent upon polymer size, with high–molecular weight (HMW) HA and low–molecular weight (LMW) HA exhibiting differing signaling properties (59). Circulating HA is reported to be in a mass range of from 1 × 10^5^ to 2 × 10^5^ Da in healthy individuals, and cleavage of HA from the EC surface results in fragments of varying mass ranges, which stimulate angiogenic and inflammatory responses (60–62). To determine whether LMW-HA degradation products are present in patients with COVID-19, we fractionated HA from plasma of healthy donors or patients with COVID-19 and determined that the majority of HA purified from healthy donor plasma is > 100 kDa, while 74.8% of plasma HA isolated from SARS-CoV-2–infected patients is present as sub–50 kDa species (Figure 5A).

We next asked whether HA released into the media of treated cells might also be present as LMW degradation products and found that cells treated with plasma from patients with COVID-19 release 55.7% of HA into the media as LMW fragments, compared with ~10.11% in cells treated with control plasma (Figure 5B). During inflammation, HA may become covalently modified with the heavy chains (HCs) of inter-α inhibitor protein (IαI), and this unique modified form of HA (HA-HC) enhances immune cell recruitment (35, 63–65) and associates with lung injury in mice and humans (66–68). Measurement of plasma HA-HC revealed that patients with COVID-19 have significantly higher levels of HA-HC compared with control subjects (Figure 5C). These data suggest that circulating HA in COVID-19 is present in the form of degradation products with inflammatory activities.

### HA fragments present in COVID-19 plasma disrupt endothelial barrier integrity.

**Figure 6:**
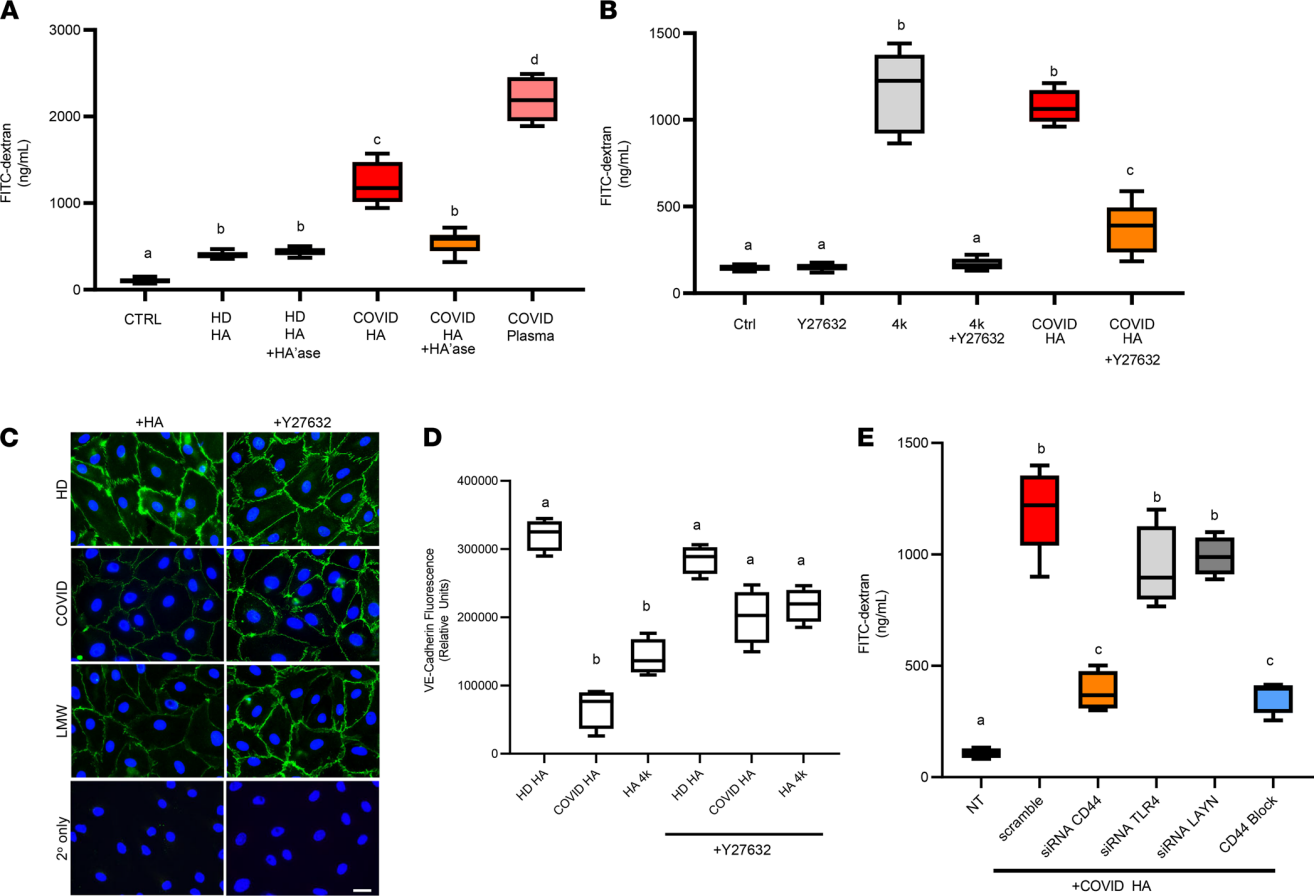
HA fragments present in COVID-19 plasma promote endothelial dysfunction. (**A**) LMVEC were seeded on permeable supports (3 μm pore size) placed into a 24-well plate and grown to confluence. Cells were treated with or without HA purified from patient plasma samples (750 ng HA) or 1.5% patient plasma for 16 hours at 37°C to induce endothelial barrier disruption. The upper chamber was replaced with FITC-conjugated dextran (1 mg/mL, 40 kDa) in PBS, and medium from the lower chamber was measured after 1 hour. (**B**) LMVECs were grown to confluence in a transwell chamber and coincubated in the presence or absence of the ROCK inhibitor Y27632 (10 μM), in the presence or absence of biosynthetic 4 kDa HA (1000 ng), or in the presence or absence of HA purified from patient plasma for 16 hours at 37°C to induce endothelial barrier disruption, and barrier function was measured as above. (**C**) LMVEC were grown on coverslips until confluency in the presence or absence of Y27632 prior to treatment with either HA purified from healthy donor plasma, COVID-19 patient plasma, or LMW-HA (4 kDa). Cells were fixed, permeabilized, and stained with an antibody against VE-cadherin. Scale bar: 20 μm. (**D**) VE-Cadherin immunostaining was quantified by using 3 independent experiments and normalized to the number of endothelial cells per field using ImageJ. (**E**) LMVECs were treated for 48 hours in the presence or absence of siRNA (10 nM) or a CD44-HA blocking antibody (KM114, 1 μg) prior to treatment with HA and measurement of barrier function. In some experiments, HA was digested with *Streptomyces* hyaluronidase (HA’ase) as a specificity control. Data are reported as mean ± SEM; *n* = 5 independent experiments of at least 4 patients each, 1-way ANOVA followed by Tukey’s multiple comparison tests. Different alphabetical superscripts are significantly different from each other; *P* < 0.05.

Circulating HA concentrations in COVID-19 patient plasma are significantly elevated compared with healthy controls (Figure 1A) and are present as LMW-HA fragments (Figure 5A) known to have barrier-disrupting effects on ECs (28). We next examined whether treatment of LMVECs with HA purified from the plasma of infected patients had a direct effect on EC barrier function. Using a transwell assay in which confluent LMVECs form a tight monolayer over a semipermeable membrane, we observed an increase in the permeability of ECs to HMW dextran after treatment with HA purified from SARS-CoV-2–infected patients, as compared with HA purified from healthy controls (Figure 6A). Importantly, the barrier-disrupting effects of HA fragments are significantly decreased by complete digestion with exogenous hyaluronidase that reduces HA to disaccharides, indicating that the increase in permeability is attributable to HA and not impurities from purification. HA purified from biological samples is a mixture of polymer lengths, and we next examined whether biosynthetic HA fragments of defined masses could reproduce the effects we observed with HA purified from COVID-19 plasma. Our data show that LMVECs incubated with LMW-HA species < 60 kDa demonstrate a size-dependent increase in barrier disruption, with HA fragments of approximately 4 kDa demonstrating the largest effect compared with control-treated cells (Supplemental Figure 5). We further analyzed cells treated with HA fragments of 4 kDa molecular weight (HA4k) for changes in the mRNA levels of HA receptors, observing a significant increase in layilin (6.3-fold) and Hyal2 (8.8-fold) expression in HA4k-treated cells compared with untreated cells, but we found no significant changes in CD44, ICAM-1, or HYAL1 (Supplemental Figure 6).

The transmembrane HA receptors CD44 and layilin both contain extracellular HA-binding domains and intracellular cytoplasmic motifs, which enable association with the actin cytoskeleton and are therefore poised to recognize and transmit signals involved in maintenance, formation, or disruption of intercellular contact. Interaction of HMW-HA by CD44 can promote barrier-enhancing effects in cultured ECs via AKT activation (69), while small HA fragments recognized by CD44 or layilin are known to disrupt cell-cell junctions in a RhoA/ROCK-dependent mechanism (70). Next, we tested whether RhoA/ROCK activation might mediate HA fragment–induced barrier disruption in lung endothelium by culturing LMVECs in the presence or absence of a ROCK inhibitor (Y27632 ) prior to treatment with either biosynthetic HA4k or COVID-19 HA fragments. As shown in Figure 6B, ROCK inhibition led to significant reduction of HA4k-induced (85.5%) and COVID-HA–induced (63.3%) permeability as compared with treatment of barrier disrupting HA fragments alone. We then examined VE-cadherin by immunostaining of LMVECs exposed to HA purified from healthy donors, patients with COVID-19, or LMW HA4k with or without pretreatment with ROCK inhibitor. We observed a loss of immunostaining for VE-cadherin in cells treated with HA purified from COVID patients or biosynthetic LMW-HA fragments compared with cells treated with HA purified from healthy donors (Figure 6, C and D), indicating that inhibition of ROCK signaling diminishes HA-mediated loss of VE-cadherin.

To determine the receptor that mediates barrier disruption in response to COVID 19–derived HA, we cultured LMVECs in the absence or presence of either scramble siRNA or siRNA targeting specific receptors with known activities regulated by LMW-HA and measured knockdown (Supplemental Figure 7). We then cultured siRNA-treated cells in the presence of HA purified from patients with COVID-19 and observed a significant loss in HA-induced barrier permeability in cells treated with CD44 siRNA, while knockdown of either layilin or TLR4 showed no statistically significant difference. We next pretreated LMVEC with an anti-CD44 antibody known to antagonize HA binding prior to incubation with HA purified from patients with COVID-19 and observed a similar inhibition of HA-induced barrier disruption to that in cells subject to CD44 knockdown (Figure 6E). Together, our data provide evidence that HA fragments present in the plasma of SARS-CoV-2–infected patients induce endothelial permeability through interaction with CD44 on the surface of LMVECs in a ROCK-dependent manner.

## Discussion

Here, we demonstrate for the first time to our knowledge that the COVID-19 cytokine storm results in aberrant degradation of endothelial glycocalyx, resulting in HA fragments capable of directly mediating endothelial dysfunction. Pathological loss of the glycocalyx during sepsis, ischemia-reperfusion injury, and other hyperinflammatory conditions is known to induce local endothelial dysfunction and release biologically active GAG and proteoglycan fragments into circulation (31, 71). Our data show that circulating GAGs are uniformly increased in COVID-19, along with corresponding activities of degradative enzymes, and are similar to those observed in patients with sepsis. Plasma levels of HA and hyaluronidase associate with SOFA scores in COVID-19 and patients with sepsis, as well as with levels of plasma cytokines known to alter HA metabolism present at elevated levels in patients with COVID-19. Importantly, we found that treatment of pulmonary microvascular ECs with plasma from patients with COVID-19 promotes HA synthesis and degradation, and this effect is due to transcriptional activation of HA synthesis and hyaluronidases. We show that the majority of HA present in COVID-19 patient plasma is present as LMW degradation products and that these HA fragments are also released into the media by cells exposed to COVID-19 plasma. Circulating GAG fragments are known to function as damage-associated molecular patterns and can affect EC activation and inflammation (28, 62). Finally, we provide mechanistic evidence that circulating HA fragments generated during SARS-CoV-2 infection can contribute to endothelial injury by inducing barrier dysfunction in a CD44- and ROCK-dependent manner.

Infection of cultured cells by measles virus, respiratory syncytial virus, and Epstein-Barr virus, as well as treatment of cells with viral mimetic and infection of mice with influenza, all promote synthesis of an inflammatory HA matrix (63, 65, 72, 73). In mice infected with influenza, excessive levels of luminal HA impairs lung function and is reversible by exogenous hyaluronidase administration (73). Recent reports indicate that HA is also elevated in the airway and respiratory secretions of patients with COVID-19 (74, 75). While our data are suggestive that the cytokine milieu associated with COVID-19 drives dysregulated HA metabolism and subsequent endothelial dysfunction, it is also possible that SARS-CoV-2 viral components themselves could induce endothelial injury. Our data share several intriguing parallels with a study of dengue virus, a hemorrhagic fever characterized by vascular leakage. The authors demonstrate that elevated HA correlates with disease severity and that dengue nonstructural protein 1 directly induces endothelial injury, release of HA-fragments, and disruption of endothelial barrier function dependent on CD44 (76). Treatment of vascular ECs with COVID-19 patient plasma has been demonstrated to induce endotheliopathy (77), and it is plausible that SARS-CoV-2 nonstructural proteins might also mediate these effects in a mechanism similar to those observed with dengue virus.

The precise mechanisms that initiate glycocalyx breakdown are not fully understood, and though we demonstrate a role for ECs in vitro, multiple cell types are likely involved during disease, including platelets that become hyperactivated in COVID-19 and are known to release HA fragments from the EC surface using HYAL-2 (35, 60). Interestingly, coagulopathy and platelet activation are also characteristic of Kawasaki disease (78). Increased serum levels of syndecan-1 and HA are known to be elevated in the acute phase of Kawasaki patients, and serum HA predicts future coronary artery lesion development, possibly implicating that similar mechanisms could occur in COVID-19 (79).

Our data raise several questions about the nature and consequence of glycocalyx degradation in critical illnesses. While cytokine release syndrome is a shared characteristic between COVID-19 and sepsis, we show that HA and HS are present at lower concentrations in COVID-19 than those observed in patients with sepsis. Plasma GAGs are removed from circulation by clearance receptors present on liver sinusoidal ECs such as stabilin-2, and injury or infection of the liver is associated with increases in circulating GAGs as in the case of hepatitis (80). Liver damage is frequently observed in patients with sepsis, but it is uncommon in COVID-19 and may explain the differences in plasma GAG concentrations observed. This explanation is supported in part by elevated bilirubin in our sepsis cohort compared with COVID-19 and by similar activities of GAG-degrading enzymes in both disease groups.

It should be noted that our study has some important limitations. First, as an observational study, we cannot conclude that the associations between glycocalyx shedding, markers of endothelial activation, and clinical outcomes are causal elements of disease progression or specific for infection with SARS-CoV-2, and we cannot exclude the possibility of remaining unmeasured potential confounders. Second, many of our patients were enrolled early in the pandemic, and we were unable to perform longitudinal examination of whether glycocalyx components exhibit a temporal dependence of shedding and if fluid resuscitation in patients with COVID-19 contributes to increased levels of released GAGs. Third, the number of patients studied represents a low sample size, especially regarding some parameters including age and race, and we were only able to examine plasma samples from patients, limiting our ability to determine the cellular source of GAG degradation. Fourth, due to the fact that many of our patients were referred to our hospital for care at various stages of disease progression, we were unable to obtain blood pressure measurements for many of our patients at the time of admission and before administration of hemodynamic support. This represents an important gap in our study, as hypertension is associated with endothelial glycocalyx dysfunction (81, 82) and is among the most common comorbidities found in hospitalized patients with COVID-19 — and because unstable blood pressure control is associated with greater risks of ICU admission and mortality in patients with COVID-19 (83, 84). Nevertheless, differences in several indices of glycocalyx degradation, including HA and hyaluronidase activity, are very pronounced and correlate well with severity of disease. Technical limitations of our study pertain to the evaluation of heparanase, where recent reports are conflicting. A small study suggests that heparanase activity is unchanged in COVID-19, despite evidence of glycocalyx injury (85), while a recent, cross-sectional report indicates that heparanase activity strongly associates with COVID-19 severity (46). However, interpretation of these data and ours may be complicated by prophylactic use of LMW heparin. In our study, 80.4% of patients with COVID-19 and 30.4% of patients with sepsis received LMW heparin, and LMW heparin treatment is associated with reduced heparanase activity in COVID-19 (46).

A growing number of studies underscore the importance of proteoglycans and GAGs of the glycocalyx as endogenous regulators of thrombosis, immune cell adhesion, and maintenance of vascular integrity, which become dysregulated in severe cases of influenza, endotoxemia, and systemic inflammation. We show that glycocalyx injury itself is a pathological manifestation capable of exacerbating disease and that the cytokine storm associated with COVID-19 is a driver of GAG-degradation by the pulmonary microvasculature. However, the spike glycoprotein of SARS-CoV-2 (86) and related coronaviruses (87, 88) contain a GAG binding motif (89) capable of mediating attachment to the cell surface via HS. It is, therefore, plausible that GAG-degrading activities could be protective mechanisms early in infection that, if unchecked, contribute to disease. The results of our investigation provide compelling evidence that prevention of enzymatic degradation of HA and other GAGs may be a treatment modality to reduce vascular permeability observed in ARDS and critically ill patients with COVID-19.

## Methods

### Study design

Patients with SARS-CoV-2 infection (*n* = 46) requiring hospitalization due to acute illness were recruited from the University of Utah Health Sciences Center in Salt Lake City (Utah, USA). We prospectively collected and analyzed data on patients with SARS-CoV-2 infection confirmed by reverse transcription PCR (RT-PCR), in accordance with current standards. ACD-anticoagulated whole blood from hospitalized patients with COVID-19 was collected from March 17–June 5, 2020. Patients with COVID-19 were recruited under study protocols approved by the IRB of the University of Utah. Healthy age- and sex-matched donors were recruited and enrolled under a separate IRB protocol. Enrollment criteria included age > 18, respiratory symptoms including cough and shortness of breath, fever, hospital admission, positive SARS-CoV-2 testing, and written informed consent. All patients with COVID-19 were hospitalized and were further stratified into non-ICU and critically ill ICU patients with COVID-19. We also enrolled critically ill patients with sepsis (*n* = 23) for comparisons. Enrollment criteria for patients with sepsis included age > 18, SEPSIS-3 criteria (a clinical consensus definition for classifying patients), and ICU admission. All patients underwent clinical investigation to identify the pathogen causing the infection and include: *Streptococcus* (11%), *Staphylococcus* (9%), *E. coli* (6%), and Influenza A/H1N1 (3%). The site or organ of the primary infection included pneumonia (34%), skin and soft tissue infection (26%), urosepsis (23%), intraabdominal infection (9%), blood (6%), or unknown (2%). Patients were a subset of subjects enrolled in previous studies (56, 90). Demographic characteristics, clinical characteristics, and illness severity data including SOFA scores are summarized in Tables 1 and 2.

### GAG purification and measurement

Plasma and media samples were proteolyzed at 55°C with 1.8 U/mL proteinase K for 16 hours to digest proteins, including those with GAG-binding function. After proteolysis, GAGs were purified stepwise by anion exchange spin columns (Thermo Fisher Scientific) using solutions of increasing concentrations of NaCl (0.15M–0.8M), were eluted, and were desalted by using 2 kDa, 50 kDa, or 100 kDa molecular weight cut off ultrafiltration devices (Thermo Fisher Scientific).

Quantification of plasma GAGs and culture mediums were measured serially using a competitive ELISA-like assay for HA (Echelon Biosciences), HS (JM403, AMSbio), and CS (LSbio). Plasma HA-HC modification was measured in nonproteolyzed plasma by in-house ELISA with HA binding protein (MilliporeSigma) and anti–rabbit IαI (DAKO, A020) antibody as previously described (91). Spike experiments to determine HS specificity were performed with the addition of 200 ng Lovenox added to patient plasma samples.

For cell treatments, HA was isolated from protease-digested plasma samples by incubation with streptavidin magnetic beads (Thermo Fisher Scientific) complexed with biotinylated versican G1 domain (Echelon Biosciences) for 24 hours at room temperature on a rocker. Beads were placed on a magnet, washed 3 times with PBS, and boiled at 95°C for 15 minutes to release bound HA. All samples were assayed for residual protein contamination by Rapid Gold BCA protein assay kit (Thermo Fisher Scientific).

### Enzyme activity assays

Enzyme activity was measured from equal volumes of plasma or media diluted in assay buffer. Hyaluronidase activity was measured as previously described (35). Heparanase activity measurements were performed using a FRET-based assay as described previously (92). Chondroitinase activity was measured using 10 μM CS-A (AMS Bio) as a substrate as described previously (93). Digestion with *Streptomyces* hyaluronidase (HA’ase, 0.1 mU/mL), recombinant human heparanase (1 μg/mL, R&D Systems), and Chondroitinase ABC (*P*. *vulgaris,* 100 ng/mL, R&D Systems) for 1 hour at 37°C was used as a reference standard to calculate units of activity. MMP2/9 and Cathepsin D activity were measured using fluorometric assay kits containing specific inhibitors according to manufacturer’s instructions (Anaspec).

### Primary cell culture

Human LMVECs were obtained from Lonza, cultured on fibronectin-coated dishes (R&D Systems), and maintained in EGM2-MV (Lonza) growth medium. Cells were incubated at 37°C in 5% CO_2_ and 95% humidity. Plasma samples were pretreated with 10 mM CaCl_2_ and defibrinated prior to addition of 1.5% plasma to culture media. Cells were incubated in the presence of plasma for 2 hours to measure changes in mRNA levels, and for 16 hours for measurement of HA or enzyme activities.

### Transwell permeability assay

LMVEC were seeded on permeable supports (3 μm pore size, Corning) placed into a 24-well plate, and grown to confluence. Cells were treated with or without HA purified from patient plasma samples (750 ng HA) or 1.5% patient plasma for 16 hours at 37°C to induce endothelial barrier disruption. The upper chamber was replaced with FITC-conjugated dextran (1 mg/mL, 40 kDa, MilliporeSigma) in PBS, and a sample of medium from the lower chamber was measured after 1 hour.

### HA receptor knockdown

Cells were grown to 70% confluency in EMG2-MV in 6-well cell culture plates and then treated with scramble, CD44, LAYN, or TLR4 siRNA (10 nM, 48 hours) using the RNAiMax transfection reagent according to the manufacturer’s instructions (Thermo Fisher Scientific). After 48 hours, cells were harvested and examined by quantitative PCR (qPCR) and Western blot. Immunodetection (1:1000 dilution) and antibody blockade (1 μg) of CD44 was performed using clone KM114 (BD Biosciences). Actin (Abcam) was used as a loading control. Quantification of knock-down was measured by densitometry analysis using ImageJ. Replicate cultures were reseeded into transwell chambers for subsequent assays.

#### Plasma preparation.

Whole blood was collected from patients using a 21 g needle vacutainer butterfly into acid/citrate/dextrose-anticoagulant. Whole blood was centrifuged (150*g,* 20 minutes, room temperature) to generate platelet-rich plasma (PRP). PRP was centrifuged at 1500*g* for 20 minutes at room temperature to produce platelet-poor plasma, and it was flash frozen in liquid nitrogen and stored at –80°C. Plasma from patients with COVID-19 or sepsis and healthy donors were isolated in a similar manner with respect to blood draws, centrifugations, and time from blood draw to freezer.

### HA size estimation

To estimate HA size ranges, human plasma samples were purified as described above. Following NaCl elution, and desalting, fractions were quantified measured by competitive HA-ELISA to determine the relative abundance of LMW HA (<50 kDa) as a function of total HA isolated from plasma specimens and culture mediums. Purified HA was quantified using an ELISA-like assay for HA (Echelon Biosciences).

### IHC

Cells were grown in 8-well chamber slides coated with fibronectin and maintained at confluency for 5 days to allow for tight junction formation. LMVECs were then treated for 16 hours in the presence or absence of purified HA or biosynthetic HA with or without addition of ROCK inhibitor Y27632. Cells were fixed with 4% paraformaldehyde, washed 3 times with PBS, and blocked for 1 hour in HBSS + 2% FBS. For VE-cadherin detection, affinity purified monoclonal antibody (1:100, Clone BV9, BioLegend) was diluted in HBSS +2% FBS and incubated at 4°C overnight in a humidified chamber. After primary exposure, cells were washed with HBSS 3 times for 5 minutes each and incubated with donkey anti–mouse Alexa Fluor 488 secondary detection antibody diluted in HBSS + 2% FBS for 1 hour. Slides were then incubated with HBSS + Hoechst 33342 (Thermo Fisher Scientific) for 10 minutes, washed with HBSS 3 times for 5 minutes each, mounted under cover glass with Prolong Glass (Thermo Fisher Scientific), and sealed with nail polish. Images were obtained using an EVOS FL Auto Cell imaging system with integrated dual camera system; system-specific software equipped with a 40×/1.42 NA objective was used. Sixteen-bit monochrome images were further analyzed, and changes were quantified using Adobe Photoshop CS6 and ImageJ (NIH).

### Plasma cytokine analysis

Plasma samples were analyzed using a Human Cytokine/Chemokine Panel I Multiplex Array (MilliporeSigma, HCYTMAG60PMX41) according to manufacturer’s instructions on a Luminex 200 instrument. Plasma samples from 14 healthy adults were used as controls for cross-comparison.

### qPCR

RNA was extracted from LMVEC and tracheal aspirates using the Direct-zol RNA kit (Zymo Research) according to the manufacturer’s protocol. The RNA was eluted into 30 μL of H_2_O. After digestion of genomic DNA by DNase, reverse transcription using the Superscript VILO kit (Thermo Fisher Scientific) was completed in accordance with the manufacturer’s instructions. The cDNA product was stored at −20°C before qPCR analysis. Validated primers with conjugated 6-carboxyfluorescein (FAM) probes for HAS1-3, HYAL1, HYAL2, CD44, Layilin, ICAM1, CD44, LAYN, TLR4, SARS-CoV-2 N1, and HPRT1 rRNA were purchased from Applied Biosystems (Invitrogen). The real-time PCR amplifications were performed in 10 μL reaction volumes that contained TaqMan gene expression Master Mix, primers and fluorogenic probes (Invitrogen), and cDNA. All reactions were performed with 4 replicate reactions using a Bio-Rad C1000 Touch Thermal Cycler with attached CFX96 Real-Time System (Bio-Rad). The real-time PCR reaction conditions were 50°C for 2 minutes and 95°C for 10 minutes, followed by 50 cycles of 95°C for 15 seconds and 60°C for 60 seconds. Changes in gene expression were calculated using the Livak (ΔΔCT) method.

### Statistics

Variables from all experiments were assessed for normality with skewness and kurtosis tests using GraphPad Prism (Anderson-Darling, D’Agostino-Pearson, Shapiro-Wilk, and Kolmogorov-Smirnov tests). For analyses comparing 2 groups, a parametric 2-tailed unpaired Student’s *t* test was used, and differences between multiple groups were calculated using 1-way ANOVA (Kruskal-Wallis test) with Dunn’s correction for multiple comparisons. When data were not normally distributed, a Mann-Whitney *U* test was used when 2 groups were analyzed. For correlations, we performed simple linear regression, Pearson correlation, or Spearman correlation. Summary statistics were used to describe the study cohort, and clinical variables are expressed as the mean ± SD or as a number and percentage where relevant. Statistical analyses were performed by using GraphPad Prism (version 8). Outliers were removed using the ROUT method with a maximum FDR set to 1%. All experiments were performed at least in triplicate, and data are represented as means ± SEM. *P* < 0.05 was considered statistically significant.

### Study approval

All studies were approved by the IRB of the University of Utah (nos. 00102638, 00093575, 0051506), and all participants or their legally authorized representatives completed written informed consent.

## Author contributions

KQ, RAM, EAM, IP, BKM, FD, EJB, RAC, and ACP designed and performed experiments; KQ, EAM, EJB, MTR, and RAC, analyzed results and made the figures; EAM, RAC, and ACP wrote the paper; and all authors reviewed and critically edited the manuscript.

## Supplementary Material

Supplemental data

## Figures and Tables

**Table 2:**
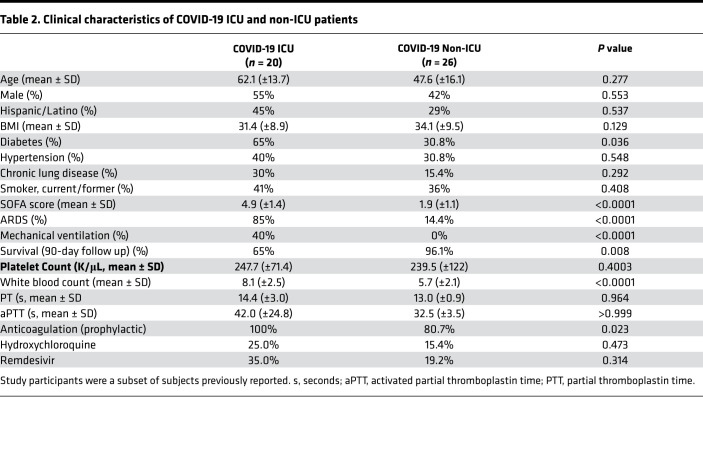
Clinical characteristics of COVID-19 ICU and non-ICU patients

**Table 1:**
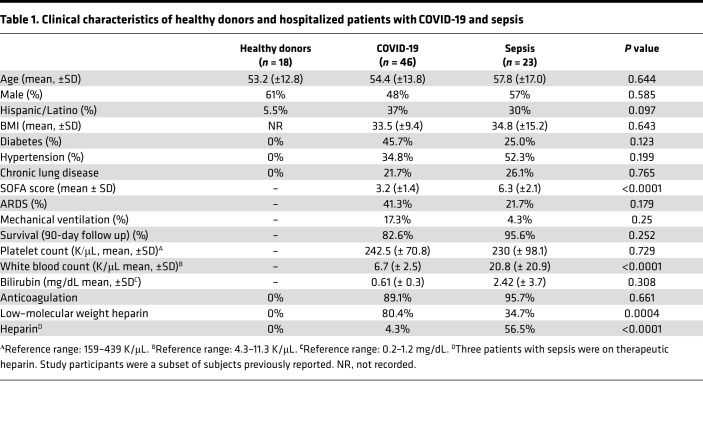
Clinical characteristics of healthy donors and hospitalized patients with COVID-19 and sepsis

## Version 1 07/27/2021

In-Press Preview

## Version 2 09/08/2021

Electronic publication
