# Supplementary material for: COVID-19 generates hyaluronan fragments that directly induce endothelial barrier dysfunction
Source: JCI Insight. 2021 Sep 8;6(17):e147472. doi: 10.1172/jci.insight.147472 (PMC8492325; doi:10.1172/jci.insight.147472)
Supplement: Supplemental data [file jciinsight-6-147472-s072.pdf]

SUPPLEMENTAL MATERIAL

Supplemental Figures

Figure S1

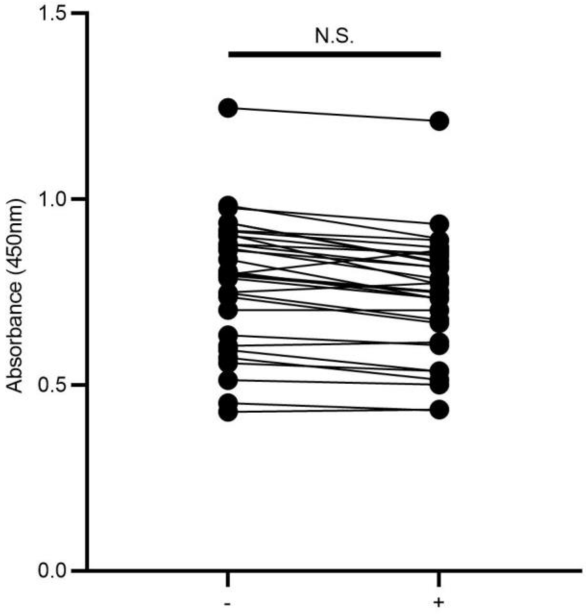

Supplemental Figure 1. **JM403-based ELISA Detection of Plasma HS Does Not Detect Exogenous Low Molecular Weight Heparin Anticoagulant Enoxaparin.** Plasma samples from healthy donors (n=10) or COVID-19 patients (n=32) were measured without (closed circles) or with (open circles) 200ng exogenous enoxaparin. Data are reported as mean ± SEM. Differences between the groups were calculated using Mann-Whitney U tests.

Figure S2

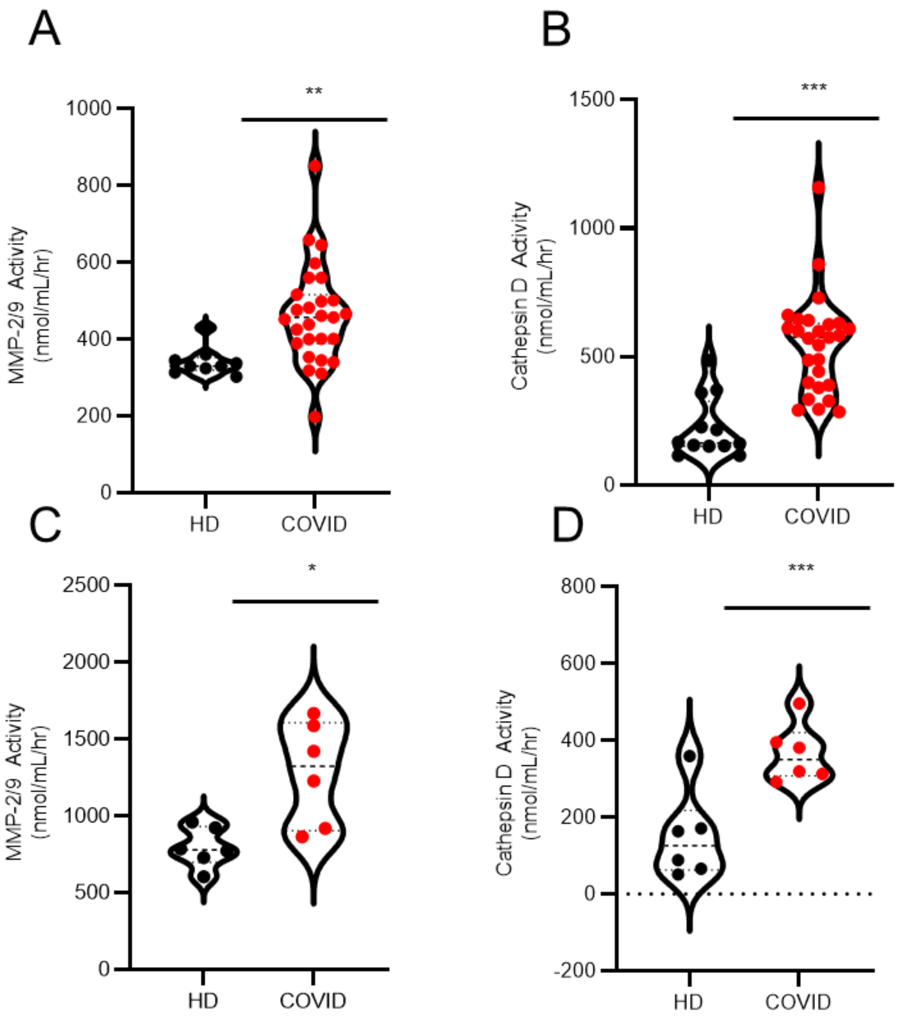

Supplemental Figure 2. **Sheddase activity is increased in COVID-19 patient plasma and released from plasma treated endothelial cells compared to healthy donor plasma.** Protease activity was measured for (A) MMP2/9 and (B) cathepsin D in healthy donors (n = 10) and COVID-19 patient plasma (n = 27). LMVECs were exposed to plasma from healthy donors or COVID-19 patients (n = 6 each). Differences between the groups were calculated using Mann-Whitney U test, **p<0.01, ***p<0.001. Sheddase activity released into the media was measured for (C) MMP2/9 and (D) cathepsin D. Data are reported as mean ± SEM. Differences between the groups were calculated using unpaired Student's t-test, 2 tailed, *p<0.05, ***p<0.001.

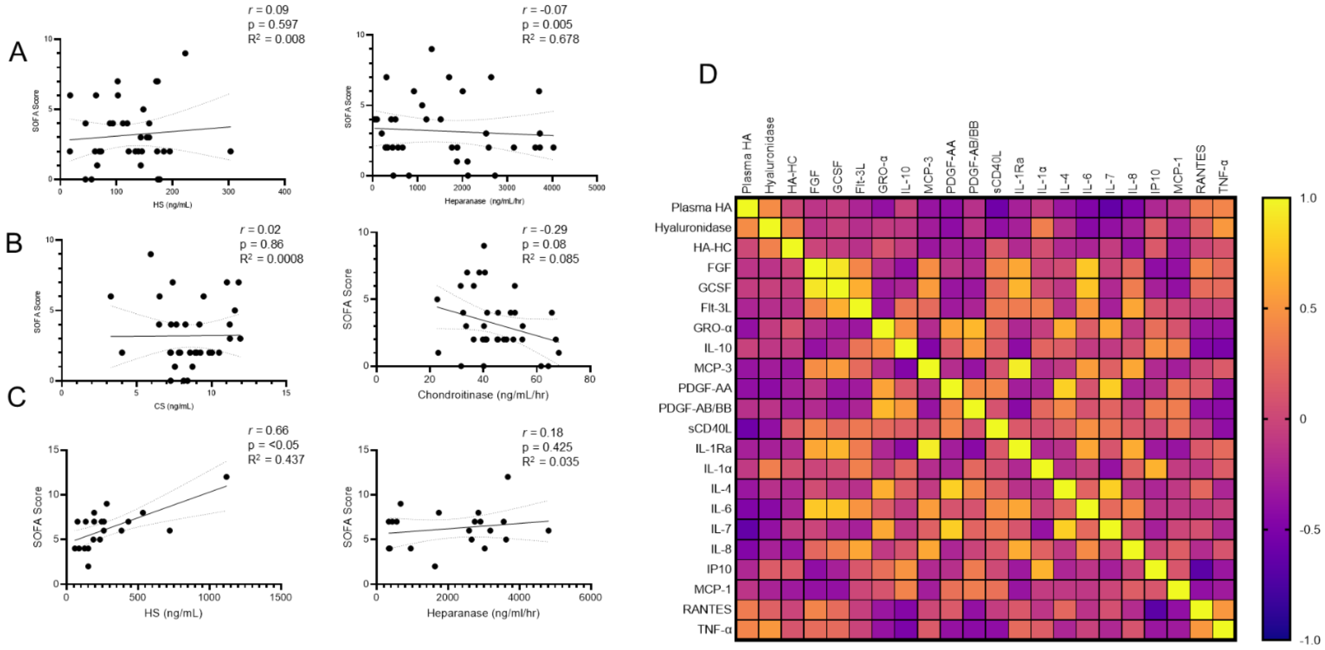

Supplemental Figure 3**. Circulating levels of HA and hyaluronidase activity correlate with clinical and inflammatory signatures of COVID-19.** Linear regression analysis of SOFA scores with (A) HS or heparanase and (B) CS or chondroitinase in COVID-19 patients (n=46). (C) Corellation analysis of SOFA scores with HS or heparanase in sepsis patients (n=23). Spearman correlation matrix (D) of plasma HA, hyaluronidase activity, and cytokine levels in healthy donors (n=18). Yellow indicates a positive correlation and purple indicates a negative correlation. Pearson correlation coefficient was used to determine the *r* value of the correlation between the 2 groups.

Figure S4.

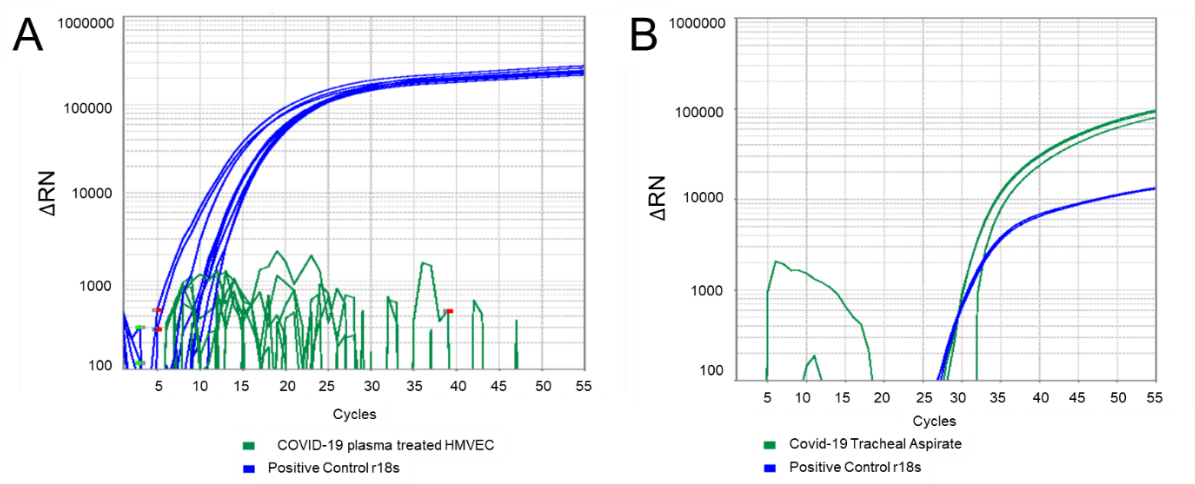

Supplemental Figure 4. **RT-PCR Analysis of LMVECs Treated With COVID-19 Plasma.** Cells were cultured in the presence or absence of COVID-19 patient plasma for 24 hours and analyzed for the presence of the SARS-CoV-2 N1 gene. (A) Representative tracing from ECs treated with COVID-19 patient plasma samples (n = 12) for SARS-CoV-2 mRNA (green) or r18s mRNA (blue) as a positive control. (B) mRNA isolated from COVID-19 patient tracheal aspirates (n = 3) served as a positive control (green) for the detection of viral mRNA.

Figure S5.

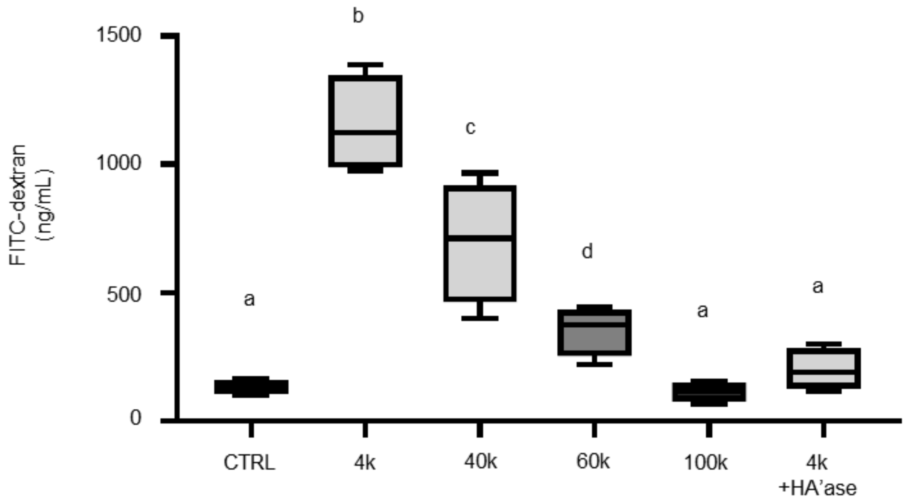

Supplemental Figure 5. **HA fragments <100 kDa induce a size-dependent disruption of the endothelial barrier permeability in LMVECs.** LMVEC were seeded on permeable supports (3 μm pore size) placed into a 24-well plate and grown to confluence. Cells were treated with or without biosynthetic HA polymers of specific sizes (1000 ng) for 16 hours at 37°C to induce endothelial barrier disruption. The upper chamber was replaced with FITC-conjugated dextran (1 mg/mL, 40kDa) in PBS and a sample of medium from the lower chamber was measured after 1 hour. In some experiments, HA treatments were pre-digested with *Streptomyces* hyaluronidase (HA'ase) as a specificity control. Data is reported as mean ± SEM; n = 5 independent experiments, statistics were calculated using a 1-way ANOVA followed by Tukey's multiple comparison tests. Different alphabetical superscripts are significantly different from each other, $p < 0.05$.

Figure S6.

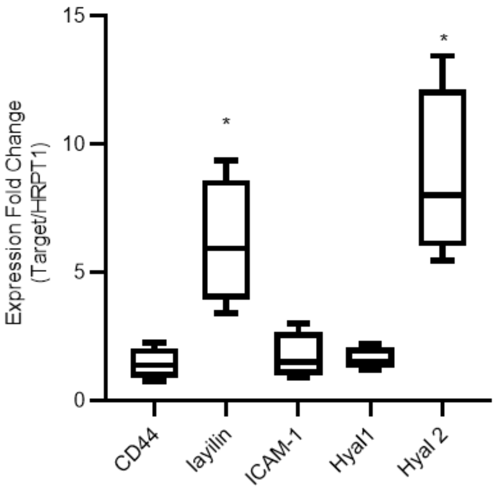

Supplemental Figure 6. **HA 4kDa treatment increases mRNA expression of layilin and Hyal-2.** LMVEC were seeded in a 6-well plate and grown to confluence. Cells were treated with or without biosynthetic HA 4kDa (1500 ng/mL) for 4 hours at 37°C. RNA was isolated from LMVEC and cDNA was made from RNA. qRT-PCR was used to measure relative mRNA expression levels of HA receptors (CD44, layilin), adhesive receptor ICAM-1, and hyaluronidases (Hyal-1, Hyal-2), normalized to housekeeping gene HPRT1. Biosynthetic HA 4kDa was pre-digested with *Streptomyces* hyaluronidase (HA'ase) as a specificity control. *p<0.05, Student's t-test, 2-tailed, n = 5 independent experiments. Data are reported as mean ± SEM.

Figure S7.

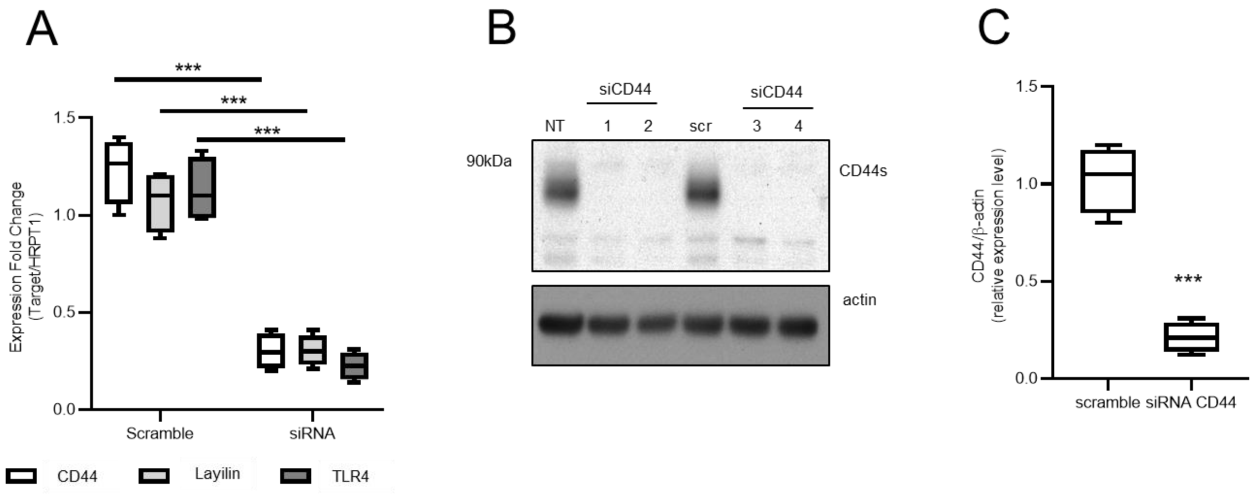

Supplemental Figure 7. **Targeted siRNA knockdown of HA-receptors.** LMVEC were seeded in a 6-well plate and grown to confluence. Cells were treated with either vehicle (NT), scrambled control siRNA (scr, 10nM), or siRNA to indicated HA receptors (10nM). (A) mRNA expression normalized to HPRT1. (B) Representative western blot analysis of CD44 protein levels from cells treated for 48hrs with 10nM CD44 siRNA. NT (no treatment), scr (scrambled control siRNA), numeric lanes indicate independent biological replicates. (C) Densitometry quantification of CD44 knock down. ***p<0.001, unpaired Student's t-test, 2-tailed, n = 5 independent experiments. Data are reported as mean ± SEM.

Supplemental Tables

A Supplemental Table 1

	Plasma HA	Hyaluronidase	HA-HC	FGF	GCSF	Flt-3L	GRO	IL-10	MCP-3	PDGF-AA	PDGF-AB/BB	sCD40L	IL-1RA	IL-1a	IL-4	IL-6	IL-7	IL-8	IP10	MCP-1	RANTES	TNFa
Plasma HA	1	0.418681979	0.640112775	0.145478721	0.283974168	-0.332446419	0.073197362	0.513474029	0.348639002	0.144464857	0.384950636	0.259672613	0.55420658	0.339773839	0.174604844	0.717066753	0.273568971	0.661657305	0.684985894	0.778090584	-0.324258103	0.62502706
Hyaluronidase	0.418681979	1	0.693359234	0.250575193	0.269596972	-0.44257053	0.50284883	0.337271388	0.066992575	0.612471773	0.603198823	0.49630061	0.188938308	0.437286546	0.511313508	0.345191527	0.411797065	0.280687591	0.503250762	0.322685192	-0.111876508	0.370579623
HA-HC	0.640112775	0.693359234	1	0.587536832	0.561555581	-0.303425481	0.185790439	0.612802653	0.330531833	0.30382428	0.598136877	0.41626888	0.531584727	0.441968278	0.280889506	0.633535318	0.338105064	0.469653362	0.667509549	0.508303604	-0.351898256	0.440642826
FGF	0.145478721	0.250575193	0.587536832	1	0.681796741	0.091850704	0.067698063	0.139376693	0.388888018	0.115546439	0.198697473	0.384843485	0.349947157	0.388895124	0.20926382	0.257850046	0.183234861	0.209464846	0.15138799	0.078192667	0.086469281	0.38078776
GCSF	0.283974168	0.269596972	0.561555581	0.681796741	1	0.11812028	0.057568014	0.206183504	0.32213346	0.073302548	0.209078506	0.351565189	0.248445878	0.499744862	0.245320281	0.179479583	0.341577577	0.322836946	0.286623235	0.205467496	0.032248324	0.432593315
Flt-3L	-0.332446419	-0.44257053	-0.303425481	0.091850704	0.11812028	1	-0.220707645	-0.230655893	0.005376261	-0.18666754	-0.145309285	-0.04366724	-0.151264301	0.021683426	-0.242190704	-0.217106	-0.099387674	0.000988902	-0.274498716	-0.155074931	0.120908257	-0.165718968
GRO	0.073197362	0.50284883	0.185790439	0.067698063	0.057568014	-0.220707645	1	-0.032493906	-0.06858982	0.591472016	0.565922189	0.542865122	-0.232329516	0.205562087	0.741005085	0.008068879	0.436177407	0.171140169	0.182290439	0.027822074	-0.161734346	0.214117078
IL-10	0.513474029	0.337271388	0.612802653	0.139376693	0.206183504	-0.230655893	-0.032493906	1	0.373366404	0.177436393	0.243986347	0.216006837	0.570384482	0.289278515	0.091361299	0.646032022	0.331109117	0.338894333	0.523203678	0.447887115	-0.370035568	0.343133589
MCP-3	0.348639002	0.066992575	0.330531833	0.388888018	0.32213346	0.005376261	-0.06858982	0.373366404	1	0.122397998	0.296344712	0.397599197	0.675483501	0.472233054	0.062937969	0.576270924	0.483597598	0.72455472	0.323784948	0.248149173	-0.121352024	0.384197082
PDGF-AA	0.144464857	0.612471773	0.30382428	0.115546439	0.073302548	-0.18666754	0.591472016	0.177436393	0.122397998	1	0.680315106	0.667101164	0.021184165	0.232974931	0.69921875	0.154067452	0.643769983	0.117206608	0.185163418	0.053097309	0.131009343	0.155417622
PDGF-AB/BB	0.384950636	0.603198823	0.598136877	0.198697473	0.209078506	-0.145309285	0.565922189	0.243986347	0.296344712	0.680315106	1	0.850396456	0.178449825	0.293505962	0.414211045	0.294636592	0.562404563	0.43325408	0.274829474	0.132119722	0.062488201	0.353488218
sCD40L	0.259672613	0.49630061	0.41626888	0.384843485	0.351565189	-0.04366724	0.542865122	0.216006837	0.397599197	0.667101164	0.850396456	1	0.285061565	0.475487476	0.496690705	0.338212664	0.658145691	0.496503224	0.28191998	0.193390304	0.018279168	0.475948282
IL-1RA	0.55420658	0.188938308	0.531584727	0.349947157	0.248445878	-0.151264301	-0.232329516	0.570384482	0.675483501	0.021184165	0.178449825	0.285061565	1	0.348822582	0.008946478	0.760414066	0.376885125	0.569624396	0.495205504	0.532451866	-0.26445315	0.533704627
IL-1a	0.339773839	0.437286546	0.441968278	0.388895124	0.499744862	0.021683426	0.205562087	0.289278515	0.472233054	0.232974931	0.293505962	0.475487476	0.348822582	1	0.471775371	0.399288915	0.610429489	0.548942486	0.711436713	0.419746602	-0.081343514	0.36834519
IL-4	0.174604844	0.511313508	0.280889506	0.20926382	0.245320281	-0.242190704	0.741005085	0.091361299	0.062937969	0.69921875	0.414211045	0.496690705	0.008946478	0.471775371	1	0.09522802	0.653507963	0.200319523	0.394302355	0.103942168	0.049371193	0.242438542
IL-6	0.717066753	0.345191527	0.633535318	0.257850046	0.179479583	-0.217106	0.008068879	0.646032022	0.576270924	0.154067452	0.294636592	0.338212664	0.760414066	0.399288915	0.09522802	1	0.334979214	0.581453768	0.590575533	0.709753753	-0.371167931	0.608728567
IL-7	0.273568971	0.411797065	0.338105064	0.183234861	0.341577577	-0.099387674	0.436177407	0.331109117	0.483597598	0.643769983	0.562404563	0.658145691	0.376885125	0.610429489	0.653507963	0.334979214	1	0.597922005	0.44072491	0.325819811	0.02694895	0.419539712
IL-8	0.661657305	0.280687591	0.469653362	0.209464846	0.322836946	0.000988902	0.171140169	0.338894333	0.72455472	0.117206608	0.43325408	0.496503224	0.569624396	0.548942486	0.200319523	0.581453768	0.597922005	1	0.520034755	0.570755038	-0.121060836	0.675110068
IP10	0.684985894	0.503250762	0.667509549	0.15138799	0.286623235	-0.274498716	0.182290439	0.523203678	0.323784948	0.185163418	0.274829474	0.28191998	0.495205504	0.711436713	0.394302355	0.590575533	0.44072491	0.520034755	1	0.701874015	-0.305792832	0.383724012
MCP-1	0.778090584	0.322685192	0.508303604	0.078192667	0.205467496	-0.155074931	0.027822074	0.447887115	0.248149173	0.053097309	0.132119722	0.193390304	0.532451866	0.419746602	0.103942168	0.709753753	0.325819811	0.570755038	0.701874015	1	-0.409632268	0.602255344
RANTES	-0.324258103	-0.111876508	-0.351898256	0.086469281	0.032248324	0.120908257	-0.161734346	-0.370035568	-0.121352024	0.131009343	0.062488201	0.018279168	-0.26445315	-0.081343514	0.049371193	-0.371167931	0.02694895	-0.121060836	-0.305792832	-0.409632268	1	-0.135483893
TNFa	0.62502706	0.370579623	0.440642826	0.38078776	0.432593315	-0.165718968	0.214117078	0.343133589	0.384197082	0.155417622	0.353488218	0.475948282	0.533704627	0.36834519	0.242438542	0.608728567	0.419539712	0.675110068	0.383724012	0.602255344	-0.135483893	1

B

	Plasma HA	Hyaluronidase	HA-HC	FGF	GCSF	Flt-3L	GRO	IL-10	MCP-3	PDGF-AA	PDGF-AB/BB	sCD40L	IL-1RA	IL-1a	IL-4	IL-6	IL-7	IL-8	IP10	MCP-1	RANTES	TNFa
Plasma HA		0.041724653	0.000323253	0.426933485	0.115230483	0.063019698	0.690536136	0.002650211	0.050514142	0.43019722	0.029585734	0.151218043	0.000997761	0.057089893	0.339180153	3.88356E-06	0.12976246	3.7288E-05	1.52658E-05	1.593E-07	0.070206336	0.0001311
Hyaluronidase	0.041724653		0.000172249	0.216955859	0.182896272	0.023570488	0.008837877	0.091997329	0.745058559	0.000881466	0.00110644	0.009914729	0.355287141	0.025489003	0.007592235	0.0841467	0.036594372	0.164847891	0.008775097	0.107878043	0.586360476	0.062371887
HA-HC	0.000323253	0.000172249		0.001271457	0.002304751	0.123908632	0.353496952	0.000678454	0.092195012	0.123390697	0.0009831	0.030791389	0.004323593	0.02098945	0.155828804	0.000388928	0.084540055	0.013447989	0.000142573	0.006787003	0.071849727	0.021423096
FGF	0.426933485	0.216955859	0.001271457		4.68285E-06	0.59418362	0.694833695	0.417529113	0.019073327	0.502179246	0.245341479	0.020469189	0.036416168	0.019070946	0.220617292	0.128937196	0.284755129	0.220164108	0.378119333	0.650337193	0.616056772	0.021953308
GCSF	0.115230483	0.182896272	0.002304751	4.68285E-06		0.492638932	0.738760831	0.227641415	0.055363405	0.670934485	0.221035622	0.035501559	0.143991319	0.001913762	0.149268907	0.294911526	0.04145807	0.054802749	0.090110719	0.229295703	0.851888221	0.00841237
Flt-3L	0.063019698	0.023570488	0.123908632	0.59418362	0.492638932		0.195827462	0.175919562	0.975174386	0.275671371	0.397782809	0.800362947	0.378513647	0.90010898	0.154693081	0.203409137	0.564134279	0.995432931	0.105197744	0.366476741	0.482410608	0.334082454
GRO	0.690536136	0.008837877	0.353496952	0.694833695	0.738760831	0.195827462		0.850772907	0.691010813	0.000145293	0.000321544	0.00062419	0.17271755	0.229076689	2.36219E-07	0.962748047	0.007829054	0.318284341	0.287287841	0.872036602	0.345996518	0.209854092
IL-10	0.002650211	0.091997329	0.000678454	0.417529113	0.227641415	0.175919562	0.850772907		0.024900433	0.300533612	0.151563668	0.205763041	0.000281202	0.087040617	0.596158755	2.08482E-05	0.048543329	0.043188998	0.001059593	0.006157972	0.026325652	0.040480284
MCP-3	0.050514142	0.745058559	0.092195012	0.019073327	0.055363405	0.975174386	0.691010813	0.024900433		0.476990712	0.07926284	0.016335162	6.18592E-06	0.003637781	0.715363317	0.000234924	0.002807835	5.84401E-07	0.054054383	0.144486354	0.480792783	0.020699971
PDGF-AA	0.43019722	0.000881466	0.123390697	0.502179246	0.670934485	0.275671371	0.000145293	0.300533612	0.476990712		5.00203E-06	8.86135E-06	0.902397847	0.171493919	2.09435E-06	0.369636736	2.27665E-05	0.496014928	0.279628148	0.758419069	0.446295061	0.365405556
PDGF-AB/BB	0.029585734	0.00110644	0.0009831	0.245341479	0.221035622	0.397782809	0.000321544	0.151563668	0.07926284	5.00203E-06		5.20465E-11	0.297736562	0.082319877	0.012021625	0.08109155	0.000356908	0.008302123	0.104762276	0.442416884	0.717313575	0.034438973
sCD40L	0.151218043	0.009914729	0.030791389	0.020469189	0.035501559	0.800362947	0.00062419	0.205763041	0.016335162	8.86135E-06	5.20465E-11		0.091954831	0.003380777	0.002060616	0.043637897	1.28514E-05	0.002069944	0.095752159	0.25843403	0.915730656	0.003345694
IL-1RA	0.000997761	0.355287141	0.004323593	0.036416168	0.143991319	0.378513647	0.17271755	0.000281202	6.18592E-06	0.902397847	0.297736562	0.091954831		0.037063069	0.958699686	7.41032E-08	0.02346473	0.000287736	0.002135528	0.000829413	0.119087353	0.000801915
IL-1a	0.057089893	0.025489003	0.02098945	0.019070946	0.001913762	0.90010898	0.229076689	0.087040617	0.003637781	0.171493919	0.082319877	0.003380777	0.037063069		0.003675251	0.0158442	7.70723E-05	0.000526502	1.15081E-06	0.010817906	0.63719845	0.02707429
IL-4	0.339180153	0.007592235	0.155828804	0.220617292	0.149268907	0.154693081	2.36219E-07	0.596158755	0.715363317	2.09435E-06	0.012021625	0.002060616	0.958699686	0.003675251		0.58063212	1.55056E-05	0.241430259	0.017329875	0.546321793	0.7749186	0.154258387
IL-6	3.88356E-06	0.0841467	0.000388928	0.128937196	0.294911526	0.203409137	0.962748047	2.08482E-05	0.000234924	0.369636736	0.08109155	0.043637897	7.41032E-08	0.0158442	0.58063212		0.045818739	0.000199956	0.000149569	1.25199E-06	0.025833766	8.1719E-05
IL-7	0.12976246	0.036594372	0.084540055	0.284755129	0.04145807	0.564134279	0.007829054	0.048543329	0.002807835	2.27665E-05	0.000356908	1.28514E-05	0.02346473	7.70723E-05	1.55056E-05	0.045818739		0.000117627	0.007139015	0.052475533	0.876020874	0.010860981
IL-8	3.7288E-05	0.164847891	0.013447989	0.220164108	0.054802749	0.995432931	0.318284341	0.043188998	5.84401E-07	0.496014928	0.008302123	0.002069944	0.000287736	0.000526502	0.241430259	0.000199956	0.000117627		0.001150533	0.000278064	0.481854038	6.2873E-06
IP10	1.52658E-05	0.008775097	0.000142573	0.378119333	0.090110719	0.105197744	0.287287841	0.001059593	0.054054383	0.279628148	0.104762276	0.095752159	0.002135528	1.15081E-06	0.017329875	0.000149569	0.007139015	0.001150533		1.84344E-06	0.069719501	0.020870237
MCP-1	1.593E-07	0.107878043	0.006787003	0.650337193	0.229295703	0.366476741	0.872036602	0.006157972	0.144486354	0.758419069	0.442416884	0.25843403	0.000829413	0.010817906	0.546321793	1.25199E-06	0.052475533	0.000278064	1.84344E-06		0.013101178	0.000101803
RANTES	0.070206336	0.586360476	0.071849727	0.616056772	0.851888221	0.482410608	0.345996518	0.026325652	0.480792783	0.446295061	0.717313575	0.915730656	0.119087353	0.63719845	0.7749186	0.025833766	0.876020874	0.481854038	0.069719501	0.013101178		0.430780116
TNFa	0.0001311	0.062371887	0.021423096	0.021953308	0.00841237	0.334082454	0.209854092	0.040480284	0.020699971	0.365405556	0.034438973	0.003345694	0.000801915	0.02707429	0.154258387	8.1719E-05	0.010860981	6.2873E-06	0.020870237	0.000101803	0.430780116	

Supplemental Table 1. **Spearman correlations for HA, hyaluronidase, HA-HC, and inflammatory cytokines in COVID-19 patients.** Spearman's ranked correlations are indicated for COVID-19 patients (n=46), R>0.5 in blue, R<-0.5 in red. Significant correlations (P<0.05) are indicated in yellow highlight. A) R values. B) P values.

A Supplemental Table 2

	Plasma HA	Hyaluronidase	HA-HC	FGF	GCSF	Flt-3L	GRO	IL-10	MCP-3	PDGF-AA	PDGF-AB/BB
Plasma HA	1	0.445459815	0.011214059	-0.127474705	-0.070088568	-0.236912752	-0.386584037	-0.038098136	-0.361681641	-0.394999038	-0.155754485
Hyaluronidase	0.445459815	1	0.383163924	-0.129553884	-0.043832771	-0.170892672	-0.081573778	-0.106618244	-0.300115747	-0.419075028	-0.133742369
HA-HC	0.011214059	0.383163924	1	-0.017583893	-0.070208984	-0.148934868	0.079563593	-0.081483327	-0.338684345	-0.271465544	-0.27851428
FGF	-0.127474705	-0.129553884	-0.017583893	1	0.912622737	0.415342188	-0.174560461	-0.378511519	0.455073789	-0.111576028	-0.198230833
GCSF	-0.070088568	-0.043832771	-0.070208984	0.912622737	1	0.641278739	-0.309285814	-0.238745721	0.532467022	-0.353191034	-0.338388769
Flt-3L	-0.236912752	-0.170892672	-0.148934868	0.415342188	0.641278739	1	-0.335569284	0.301898367	0.239784203	-0.313572338	-0.242336509
GRO-α	-0.386584037	-0.081573778	0.079563593	-0.174560461	-0.309285814	-0.335569284	1	0.469653163	-0.188209012	0.583375729	0.692049966
IL-10	-0.038098136	-0.106618244	-0.081483327	-0.378511519	-0.238745721	0.301898367	0.469653163	1	-0.467002335	0.124428495	0.519706178
MCP-3	-0.361681641	-0.300115747	-0.338684345	0.455073789	0.532467022	0.239784203	-0.188209012	-0.467002335	1	-0.179882376	-0.366264349
PDGF-AA	-0.394999038	-0.419075028	-0.271465544	-0.111576028	-0.353191034	-0.313572338	0.583375729	0.124428495	-0.179882376	1	0.424111942
PDGF-AB/BB	-0.155754485	-0.133742369	-0.27851428	-0.198230833	-0.338388769	-0.242336509	0.692049966	0.519706178	-0.366264349	0.424111942	1
sCD40L	-0.572847224	-0.393186779	0.136525921	0.387621464	0.240965946	0.328855862	0.235229878	0.148982814	0.073834026	0.418006435	0.074640268
IL-1Ra	-0.272477259	-0.274641055	-0.201047074	0.603804226	0.673781882	0.350043639	-0.217730799	-0.413132944	0.944248938	-0.287640171	-0.441325208
IL-1α	-0.091575231	0.367695301	0.055779365	-0.019463784	0.036643499	0.37392224	-0.058317288	0.200587518	-0.177631721	-0.235209976	0.220368919
IL-4	-0.341776974	-0.159386926	-0.210988792	0.042035453	-0.107013914	-0.15758225	0.642688097	0.147631014	-0.082901372	0.809326641	0.417680166
IL-6	-0.48672547	-0.442246409	-0.240202446	0.771773436	0.751099704	0.490429722	0.145968812	-0.086202264	0.543807063	0.086039761	0.076658763
IL-7	-0.633954516	-0.384986507	-0.067180503	-0.01950527	-0.210870284	-0.273507748	0.605394857	-0.090636261	0.173468717	0.800778094	0.193909016
IL-8	-0.476635941	-0.263830309	-0.353157335	0.219084844	0.358023065	0.598279601	-0.096587353	0.089429131	0.601972999	-0.234300092	-0.022229834
IP10	-0.220456294	0.137909699	0.123034456	-0.410117105	-0.273904512	0.107168747	0.265229807	0.489325798	-0.205853442	-0.163718922	0.345165981
MCP-1	-0.124542581	-0.252153299	-0.324690778	-0.383273099	-0.387391401	-0.002521863	0.084938376	0.394477225	-0.288210461	0.281647841	0.360506314
RANTES	0.349931295	0.173677741	-0.017470034	0.397440598	0.249236473	-0.055042767	-0.34387662	-0.4649094	0.004385924	0.128923404	-0.385267907
TNF-α	0.410259923	0.519561553	0.093998496	0.249500765	0.24551292	-0.025804457	-0.370878481	-0.511215807	0.132530146	-0.375669976	-0.427892347

	sCD40L	IL-1RA	IL-1a	IL-4	IL-6	IL-7	IL-8	IP10	MCP-1	RANTES	TNFa
Plasma HA	-0.572847224	-0.272477259	-0.091575231	-0.341776974	-0.48672547	-0.633954516	-0.476635941	-0.220456294	-0.124542581	0.349931295	0.410259923
Hyaluronidase	-0.393186779	-0.274641055	0.367695301	-0.159386926	-0.442246409	-0.384986507	-0.263830309	0.137909699	-0.252153299	0.173677741	0.519561553
HA-HC	0.136525921	-0.201047074	0.055779365	-0.210988792	-0.240202446	-0.067180503	-0.353157335	0.123034456	-0.324690778	-0.017470034	0.093998496
FGF	0.387621464	0.603804226	-0.019463784	0.042035453	0.771773436	-0.01950527	0.219084844	-0.410117105	-0.383273099	0.397440598	0.249500765
GCSF	0.240965946	0.673781882	0.036643499	-0.107013914	0.751099704	-0.210870284	0.358023065	-0.273904512	-0.387391401	0.249236473	0.24551292
Flt-3L	0.328855862	0.350043639	0.37392224	-0.15758225	0.490429722	-0.273507748	0.598279601	0.107168747	-0.002521863	-0.055042767	-0.025804457
GRO-α	0.235229878	-0.217730799	-0.058317288	0.642688097	0.145968812	0.605394857	-0.096587353	0.265229807	0.084938376	-0.34387662	-0.370878481
IL-10	0.148982814	-0.413132944	0.200587518	0.147631014	-0.086202264	-0.090636261	0.089429131	0.489325798	0.394477225	-0.4649094	-0.511215807
MCP-3	0.073834026	0.944248938	-0.177631721	-0.082901372	0.543807063	0.173468717	0.601972999	-0.205853442	-0.288210461	0.004385924	0.132530146
PDGF-AA	0.418006435	-0.287640171	-0.235209976	0.809326641	0.086039761	0.800778094	-0.234300092	-0.163718922	0.281647841	0.128923404	-0.375669976
PDGF-AB/BB	0.074640268	-0.441325208	0.220368919	0.417680166	0.076658763	0.193909016	-0.022229834	0.345165981	0.360506314	-0.385267907	-0.427892347
sCD40L	1	0.123079548	0.054511959	0.172651224	0.462769777	0.305409129	0.125428965	0.019442747	0.28074691	-0.072846826	-0.407500236
IL-1Ra	0.123079548	1	-0.208930011	-0.148129258	0.595641559	0.090493662	0.627650047	-0.318564174	-0.361812913	0.121113702	0.241005111
IL-1α	0.054511959	-0.208930011	1	-0.161016288	-0.032664778	-0.370038075	0.183459554	0.657948286	-0.079658545	-0.119628471	0.150844484
IL-4	0.172651224	-0.148129258	-0.161016288	1	0.192497029	0.807763219	-0.100033519	-0.233859939	-0.100394834	0.217040123	-0.233405264
IL-6	0.462769777	0.595641559	-0.032664778	0.192497029	1	0.238982517	0.437910267	-0.129011259	-0.111582343	-0.137462259	-0.041185168
IL-7	0.305409129	0.090493662	-0.370038075	0.807763219	0.238982517	1	0.05913896	-0.28051553	-0.005406287	0.060073255	-0.2207496
IL-8	0.125428965	0.627650047	0.183459554	-0.100033519	0.437910267	0.05913896	1	-0.012791334	0.157169164	-0.187998812	0.087124385
IP10	0.019442747	-0.318564174	0.657948286	-0.233859939	-0.129011259	-0.28051553	-0.012791334	1	0.100414037	-0.678391262	-0.34819471
MCP-1	0.28074691	-0.361812913	-0.079658545	-0.100394834	-0.111582343	-0.005406287	0.157169164	0.100414037	1	-0.39178003	-0.314688149
RANTES	-0.072846826	0.121113702	-0.119628471	0.217040123	-0.137462259	0.060073255	-0.187998812	-0.678391262	-0.39178003	1	0.512111352
TNF-α	-0.407500236	0.241005111	0.150844484	-0.233405264	-0.041185168	-0.2207496	0.087124385	-0.34819471	-0.314688149	0.512111352	1

B

	Plasma HA	Hyaluronidase	HA-HC	FGF	GCSF	Flt-3L	GRO	IL-10	MCP-3	PDGF-AA	PDGF-AB/BB
Plasma HA		0.083774075	0.968360413	0.6640876	0.811812331	0.414781423	0.172125836	0.897116211	0.203845473	0.162178793	0.594927599
Hyaluronidase	0.083774075		0.158617329	0.658908895	0.881722404	0.55913245	0.781606799	0.716775196	0.297171957	0.135826593	0.648519668
HA-HC	0.968360413	0.158617329		0.952424373	0.811494304	0.611343124	0.786874085	0.781843621	0.236212399	0.347818907	0.334940707
FGF	0.6640876	0.658908895	0.952424373		5.31059E-06	0.139710503	0.550598995	0.182032444	0.102044267	0.704134339	0.496915893
GCSF	0.811812331	0.881722404	0.811494304	5.31059E-06		0.013447454	0.281921739	0.411072796	0.049972284	0.215449607	0.236647752
Flt-3L	0.414781423	0.55913245	0.611343124	0.139710503	0.013447454		0.240825309	0.294170624	0.408979261	0.274954423	0.403857421
GRO-α	0.172125836	0.781606799	0.786874085	0.550598995	0.281921739	0.240825309		0.090186303	0.519344648	0.028529263	0.006097802
IL-10	0.897116211	0.716775196	0.781843621	0.182032444	0.411072796	0.294170624	0.090186303		0.092267015	0.671700105	0.056808999
MCP-3	0.203845473	0.297171957	0.236212399	0.102044267	0.049972284	0.408979261	0.519344648	0.092267015		0.538317168	0.19774978
PDGF-AA	0.162178793	0.135826593	0.347818907	0.704134339	0.215449607	0.274954423	0.028529263	0.671700105	0.538317168		0.130701463
PDGF-AB/BB	0.594927599	0.648519668	0.334940707	0.496915893	0.236647752	0.403857421	0.006097802	0.056808999	0.19774978	0.130701463	
sCD40L	0.032258567	0.164288406	0.641647859	0.170878646	0.40660364	0.250952663	0.418201425	0.611227107	0.801933134	0.136930954	0.799810094
IL-1Ra	0.3459539	0.341983757	0.490695115	0.022222227	0.008240929	0.219853842	0.454604248	0.142043731	3.84195E-07	0.318670636	0.114173877
IL-1α	0.755533112	0.19587039	0.8497836	0.947344181	0.901026535	0.187824617	0.843024452	0.491707736	0.54349659	0.418241957	0.449019406
IL-4	0.231686764	0.586256088	0.469029803	0.886543118	0.71576383	0.590557921	0.013179613	0.614501348	0.778132856	0.000453777	0.137269338
IL-6	0.077565285	0.113331792	0.408137662	0.001222531	0.001958925	0.075001068	0.61853659	0.769512146	0.044416788	0.769935972	0.794500546
IL-7	0.014907043	0.174057908	0.819500849	0.94723209	0.469285318	0.344059986	0.021779552	0.75797118	0.553133209	0.000578786	0.506532712
IL-8	0.084862517	0.362071835	0.215496469	0.451733529	0.208796159	0.023812517	0.742555204	0.761108608	0.022740212	0.420097136	0.939872678
IP10	0.448835015	0.638241555	0.675193653	0.145270518	0.343332317	0.715368183	0.359435894	0.075758873	0.480163071	0.575981795	0.226789244
MCP-1	0.671414472	0.38447174	0.257363388	0.176145686	0.171154718	0.993173311	0.772810086	0.162784396	0.317669091	0.329302563	0.205427731
RANTES	0.220012076	0.552647627	0.952732118	0.15936479	0.390179338	0.851747171	0.228644907	0.093933266	0.988127547	0.660477772	0.173716564
TNF-α	0.145116622	0.05689015	0.749250833	0.389660341	0.39753003	0.930223988	0.191730522	0.061716056	0.651520491	0.185605046	0.126941476

	sCD40L	IL-1RA	IL-1a	IL-4	IL-6	IL-7	IL-8	IP10	MCP-1	RANTES	TNFa
Plasma HA	0.032258567	0.3459539	0.755533112	0.231686764	0.077565285	0.014907043	0.084862517	0.448835015	0.671414472	0.220012076	0.145116622
Hyaluronidase	0.164288406	0.341983757	0.19587039	0.586256088	0.113331792	0.174057908	0.362071835	0.638241555	0.38447174	0.552647627	0.05689015
HA-HC	0.641647859	0.490695115	0.8497836	0.469029803	0.408137662	0.819500849	0.215496469	0.675193653	0.257363388	0.952732118	0.749250833
FGF	0.170878646	0.022222227	0.947344181	0.886543118	0.001222531	0.94723209	0.451733529	0.145270518	0.176145686	0.15936479	0.389660341
GCSF	0.40660364	0.008240929	0.901026535	0.71576383	0.001958925	0.469285318	0.208796159	0.343332317	0.171154718	0.390179338	0.39753003
Flt-3L	0.250952663	0.219853842	0.187824617	0.590557921	0.075001068	0.344059986	0.023812517	0.715368183	0.993173311	0.851747171	0.930223988
GRO-α	0.418201425	0.454604248	0.843024452	0.013179613	0.61853659	0.021779552	0.742555204	0.359435894	0.772810086	0.228644907	0.191730522
IL-10	0.611227107	0.142043731	0.491707736	0.614501348	0.769512146	0.75797118	0.761108608	0.075758873	0.162784396	0.093933266	0.061716056
MCP-3	0.801933134	3.84195E-07	0.54349659	0.778132856	0.044416788	0.553133209	0.022740212	0.480163071	0.317669091	0.988127547	0.651520491
PDGF-AA	0.136930954	0.318670636	0.418241957	0.000453777	0.769935972	0.000578786	0.420097136	0.575981795	0.329302563	0.660477772	0.185605046
PDGF-AB/BB	0.799810094	0.114173877	0.449019406	0.137269338	0.794500546	0.506532712	0.939872678	0.226789244	0.205427731	0.173716564	0.126941476
sCD40L		0.675080554	0.853162654	0.555034061	0.095658202	0.288311514	0.669196654	0.947401022	0.330918086	0.804534412	0.148109592
IL-1Ra	0.675080554		0.473478205	0.613293755	0.02460136	0.758341627	0.016257543	0.266970637	0.20366923	0.680017143	0.40652503
IL-1α	0.853162654	0.473478205		0.582383039	0.911732472	0.192818034	0.530129604	0.01053348	0.786625091	0.683754753	0.606728927
IL-4	0.555034061	0.613293755	0.582383039		0.509692929	0.000474842	0.733668549	0.420996074	0.732738597	0.456071983	0.421925701
IL-6	0.095658202	0.02460136	0.911732472	0.509692929		0.410594941	0.117332783	0.660259078	0.704118283	0.639342251	0.888825008
IL-7	0.288311514	0.758341627	0.192818034	0.000474842	0.410594941		0.840838256	0.331333708	0.985365726	0.838353701	0.448216315
IL-8	0.669196654	0.016257543	0.530129604	0.733668549	0.117332783	0.840838256		0.9653829	0.591544372	0.519819874	0.767108278
IP10	0.947401022	0.266970637	0.01053348	0.420996074	0.660259078	0.331333708	0.9653829		0.73268918	0.007652352	0.222467042
MCP-1	0.330918086	0.20366923	0.786625091	0.732738597	0.704118283	0.985365726	0.591544372	0.73268918		0.16593828	0.273157687
RANTES	0.804534412	0.680017143	0.683754753	0.456071983	0.639342251	0.838353701	0.519819874	0.007652352	0.16593828		0.061184618
TNF-α	0.148109592	0.40652503	0.606728927	0.421925701	0.888825008	0.448216315	0.767108278	0.222467042	0.273157687	0.061184618	

Supplemental Table 2. **Spearman correlations for HA, hyaluronidase, HA-HC, and inflammatory cytokines in healthy donors.** Spearman's ranked correlations are indicated for healthy donors (n=18), R>0.5 in blue, R<-0.5 in red. Significant correlations (P<0.05) are indicated in yellow highlight. A) R values. B) P values.
